# Release of 50 new, drug-like compounds and their computational target predictions for open source anti-tubercular drug discovery

**DOI:** 10.1371/journal.pone.0142293

**Published:** 2015-12-07

**Authors:** María Jose Rebollo-Lopez, Joël Lelièvre, Daniel Alvarez-Gomez, Julia Castro-Pichel, Francisco Martínez-Jiménez, George Papadatos, Vinod Kumar, Gonzalo Colmenarejo, Grace Mugumbate, Mark Hurle, Vanessa Barroso, Rob J. Young, María Martinez-Hoyos, Rubén González del Río, Robert H. Bates, Eva Maria Lopez-Roman, Alfonso Mendoza-Losana, James R. Brown, Emilio Alvarez-Ruiz, Marc A. Marti-Renom, John P. Overington, Nicholas Cammack, Lluís Ballell, David Barros-Aguire

**Affiliations:** 1 Diseases of the Developing World, GlaxoSmithKline, Tres Cantos, Madrid, Spain; 2 Genome Biology Group, Centre Nacional d’Anàlisi Genòmica (CNAG), Barcelona, Spain; 3 Gene Regulation Stem Cells and Cancer Program, Centre for Genomic Regulation (CRG), Barcelona, Spain; 4 European Molecular Biology Laboratory–European Bioinformatics Institute (EMBL-EBI), Hinxton, Cambridge, United Kingdom; 5 Computational Biology, Quantitative Sciences, GlaxoSmithKline, Collegeville, Pennsylvania, United States of America; 6 Centro de Investigación Básica, CSci Computational Chemistry, GlaxoSmithKline, Tres Cantos, Madrid, Spain; 7 CSC Medicinal Chemistry, Medicines Research Centre, GlaxoSmithKline, Stevenage, Hertfordshire, United Kingdom; 8 Institució Catalana de Recerca i Estudis Avançats (ICREA), Barcelona, Spain; 9 Centro de Investigación Básica, Platform Technology & Science, GlaxoSmithKline, Tres Cantos, Madrid, Spain; University of Delhi, INDIA

## Abstract

As a follow up to the antimycobacterial screening exercise and the release of GSK´s first Tres Cantos Antimycobacterial Set (TCAMS-TB), this paper presents the results of a second antitubercular screening effort of two hundred and fifty thousand compounds recently added to the GSK collection. The compounds were further prioritized based on not only antitubercular potency but also on physicochemical characteristics. The 50 most attractive compounds were then progressed for evaluation in three different predictive computational biology algorithms based on structural similarity or GSK historical biological assay data in order to determine their possible mechanisms of action. This effort has resulted in the identification of novel compounds and their hypothesized targets that will hopefully fuel future TB drug discovery and target validation programs alike.

## Introduction

Although the Millennium Development Goal (MDG) target to halt and reverse the Tuberculosis (TB) epidemic by 2015 has been achieved, the global burden of disease remains enormous. The World Health Organization (WHO) estimates that about one third of the world’s population could be latently infected with Tuberculosis. Although the vast majority will not go on to develop TB, in 2012 there were 8.6 million new cases of active disease, 1.3 million of which resulted in death attributable to TB [1].

More worryingly, the increasing prevalence of Multi Drug Resistant (MDR) and Extensively Drug Resistant (XDR) TB highlights the shortcomings of the present therapeutic options against the disease [1]. According to WHO, in 2013, there were an estimated 480 000 new cases of MDR-TB worldwide, 9% of which were found to be practically untreatable XDR-TB. In 2014, XDR-TB has already been reported by 100 countries. Therefore, there is still an urgent need for new drugs with novel mechanisms of action, able to treat both MDR/XDR and drug sensitive TB patients in a cost effective way. To not tackle this challenge head on now will be at our own future peril.

To stimulate community-based research efforts towards the discovery of novel TB therapeutics, and as a follow-up to our previous release of 177 compounds into the public domain [2] as promising starting points for new TB medicines, we have recently screened the latest chemical diversity available within GSK compound collections and identified 50 novel, non-cytotoxic, high quality chemical starting points active against replicating *Mycobacterium tuberculosis*. The presentation of this data has been complemented with a multipronged computational analysis to predict the possible biological targets of each one of these molecules.

## Materials and Methods

### HTS ATP assay

While the resazurin-based method was a reliable way to test the phenotypic activity of antitubercular compounds, it was unfortunately unsuitable for HTS campaigns given the low signal-to-noise ratio and the frequent interference of fluorescent compounds. As an alternative to a resazurin-based readout, we used a commercially available system based on ATP measurement (BacTiter-Glo, Promega). This assay measured the effect of the compounds on bacterial growth by determining the amount of ATP per well, which is proportional to the number of living bacteria. The reagent caused bacterial cell lysis and generated a luminescent signal proportional to the amount of ATP present and thus to the number of viable cells in culture. The assay relied on the activity of a thermostable luciferase and on the properties of a buffer formulation for extracting ATP from bacteria.

### Single Shot inhibition assay

#### *Mycobacterium bovis* BCG str. Pasteur 1173P2 (BCG)

Bacterial inocula were grown for 4–5 days in Middlebrook 7H9 medium (Difco cat. # 271310) with glucose as carbon source. The culture medium contained per liter: 4.7 g Middlebrook 7H9 powder, 5 g albumin, 1 g glucose, 0.85 g NaCl, and 0.25 g Tween 80. The solution was sterilized by filtration through a 0.2 mm filter. The HTS assay was carried out in 1536-well sterile plates (Greiner, 782074). The screening compounds were added to the plates as a 50 nL solution in neat DMSO (Sigma, D8418) prior to addition of the assay components by using an Echo 555 instrument (Labcyte Inc). The assay plates were subsequently filled with 5 μL of the bacterial solution (adjusted to 10^5^ bacteria per mL) using a Multidrop Combi NL instrument (Thermo Fischer Scientific Inc.). Inoculated plates were stacked in groups of 7–8 plates, with the top plate covered with a sterile lid. Plates were carefully wrapped with aluminum foil to prevent evaporation and allowed to incubate at 37°C at 80% relative humidity for seven days. After the incubation period, plates were removed from the incubator and allowed to equilibrate at room temperature. Freshly reconstituted BacTiter-Glo (5 μL, Promega) was added to each well using the Multidrop Combi. After standing at room temperature for 7–8 min, the luminescence signal was quantified with an Acquest reader (Molecular Devices) in the focused luminescence mode. Every assay plate contained two columns of negative controls (control 1) with DMSO, which correspond to 100% activity reactions (maximum luminescence), and two columns of positive controls (control 2) in which 100% inhibition was reached by adding a known inhibitor (2 μM rifampicin as standard; bacterial growth completely inhibited). These controls were used to monitor assay quality through determination of Z´ as well as for normalizing the data on a per-plate basis. The effect of a given compound is calculated as: % Inhib. = 100 x [(data—ctrl 1)/(ctrl2—ctrl 1)].

#### *Mycobacterium tuberculosis* H37Rv (*M*. *tuberculosis*)

For *M*. *tuberculosis*, the HTS assay was carried out in sterile 384-well white microtest plates TC surface (353988 BC Falcon). 250 nL of screening compound were added to the plates as a solution in neat DMSO. The inoculum was standardized to 10^7^ CFU/mL by measuring the OD at 600nm (an OD600 = 0.125 is equivalent to 10^7^ CFU/mL) and then diluted 1 in 100 (10^5^ CFU/mL) in 4.7 g Middlebrook 7H9 powder, 5 g albumin, 1 g glucose, 0.85 g NaCl, and 0.025% Tyloxapol (Sigma T8761). 25 μL of the 10^5^ CFU/mL solution were dispensed in all 384w compound plates. Every assay plate contained one column of negative control (control 1, 6^th^ column) with neat DMSO and one column of positive control (control 2, 18^th^ column) in which 100% inhibition was reached by adding a known inhibitor (0.1 mg/mL of rifampicin, Sigma R3501). The incubation was as described previously [2]. This time, 10 μL of reconstituted BacTiter-Glo™ Microbial Cell viability Assay (Promega, G8231) reagent was added to each well and the plate was left 30 min at room temperature. The luminescence was measured using the Spectramax M5 (Molecular Devices) with integration time 250 mseconds (endpoint).

### pIC_50_ ATP assay (*M*. *bovis* BCG Pasteur and *M*. *tuberculosis* H37Rv)

The assay was performed in 384 well plates for *M*. *tuberculosis* H37Rv and in 1536 well plates for *M*. *bovis* BCG Pasteur. For each compound, 11 two-fold dilutions were done in DMSO (final concentration 1%). The controls were as the ones used for the *M*. *tuberculosis H37Rv* Single Shot ATP assay. The method used (inocula, incubation, measurement) is the same as in the *M*. *tuberculosis* H37Rv Single Shot ATP assay, maintaining 8 min incubation, once the BacTiter-Glo™ is added, in the case of *M*. *bovis BCG Pasteur* and 30 min for *M*. *tuberculosis H37Rv*.

The effect of a given compound was calculated as % inhibition at single shot or pIC_50_ (ActivityBase, ID Business Solutions Limited). Zprime lower limit had been established at 0.4. Plates with Zprime values below this cutoff were rejected.

### Statistical analysis for HTS

Statistical cutoffs were obtained as the mean plus 3 standard deviations calculated with a robust algorithm [3] from the population of growth inhibitions; compounds above the statistical cutoffs were deemed to have significant inhibition compared to the majority of the compounds that had inhibitions within the noise. A cutoff was calculated for each batch of plates tested in one day. Previously the plates were corrected for the presence of patterns when necessary by using an in-house developed plate pattern recognition and fixing algorithm [4].

### Pattern Correction

The plates that display gradient patterns were fixed with the Pattern Recognition & Fixing Algorithm. The algorithm corrects the responses by calculating a robust 2D running median across the wells and performs a weighted subtraction from the original responses such as it leaves unmodified the “outlier” responses. Plates with VEP (variance explained by pattern) > 0.35 were deemed in need of pattern fixing.

### Activity against Non-replicating *Mycobacterium tuberculosis*

Non-Replicating (NR) conditions were induced in Sauton’s-based minimal containing 0.05% KH_2_PO_4_, 0.05% MgSO_4_, 0.005% ferric ammonium citrate, 0.00001% ZnSO_4_ and 0.01% NH_4_Cl supplemented with 0.05% butyrate, 0.5% BSA, 0.085% NaCl and 0.02% tyloxapol. pH was set to 5.0 with 2N NaOH and NaNO_2_ was added from freshly prepared 1M stock (in distilled H_2_O) to a final concentration of 0.5mM. NR conditions also included 1% O_2_ and 5% CO_2_.

Assay conditions: 150 nL of each compound at 1mM concentration (in 100% DMSO) dispensed in 384 well plates and stored at -20°C. Bacterial pellets obtained from log-phase *M*. *tuberculosis* H37Rv grown in roller bottles at 37°C and 20% O_2_ were washed twice with phosphate buffer saline (PBS; Difco), which had 0.02% tyloxapol (PBS-Tyloxapol). Bacterial suspension with an OD of 0.1 at 580 nm was then prepared in NR medium and NaNO_2_ added fresh for a final concentration of 0.5 mM. 15 μL of this suspension was dispensed in to each well of the compound plate. Plates were incubated for 3 days at 37°C in oxygen-controlled incubators at 1% O_2_ and 5% CO_2_. 60 μL of complete 7H9 medium was added to each well after NR exposure and the plates incubated at 37°C with 21% O2 and 5% CO_2_ to allow outgrowth of bacteria. OD was read after 7 days using a microplate reader.

### *M*. *tuberculosis* inhibition assay (MABA)–H37Rv and resistant strains

The measurement of the minimum inhibitory concentration (MIC) for each tested compound was performed in 96-well flat-bottom polystyrene microtiter plates. Ten twofold drug dilutions in neat DMSO starting at 5 mM were performed. These drug solutions (5 μL) were added to 95 μL Middlebrook 7H9 medium (lines A-H, rows 1–10 of the plate layout). Isoniazid was used as a positive control; eight twofold dilutions of isoniazid starting at 1.2 mM were prepared, and this control curve (5 μL) was added to 95 μL Middlebrook 7H9 medium (row 11, lines A-H). Neat DMSO (5 μL) was added to row 12 (growth and blank controls). The inoculums were standardized to ~1x10^7^ CFUmL^-1^ and diluted 1:100 in Middlebrook 7H9 broth (Middlebrook ADC enrichment, a dehydrated culture medium which supports growth of mycobacterial species, available from Becton–Dickinson, cat. # 211887), to produce the final inoculum of H37Rv strain (ATCC25618) and resistant clinical isolates to isoniazid and rifampicin. This inoculum (100 mL) was added to the entire plate except G-12 and H-12 wells (blank controls). All plates were placed in a sealed box to prevent drying out of the peripheral wells and were incubated at 37°C without shaking for six days. A resazurin solution was prepared by dissolving one tablet of resazurin (VWR International Ltd., Resazurin Tablets for Milk Testing, cat.# 330884Y’) in 30 mL sterile phosphate-buffered saline (PBS). Of this solution, 25 μL were added to each well. Fluorescence was measured (Spectramax M5, Molecular Devices; lex 530 nm, lem 590 nm, cutoff 570 nm) after 48 h to determine the MIC value.

### Intracellular assay

M. tuberculosis H37Rv containing the Photinus pyralis luciferase gene (Hygromicin resistant plasmid) was grown in 7H9 suplemented with 10% ADC and 0.05% Tyloxapol until the OD600 is 0.5–0.8. We divided 160 ml of culture in 4 tubes of 50 ml each and pelleted at 2860g for 10 min. 10 glass beads (4mm) were added in order to disperse the bacterial pellet of each tube by shaking for 60 seconds. Then 6 ml of fresh RPMI media were added and leave on the bench for 5 min. Carefully we collected 5 ml of the supernatant and discard the rest. The supernatants of 4 tubes were collected into the same sterile tube and centrifuged at 402g for 5 minutes to avoid any remaining clumps. This dispersed bacterial suspension was diluted into RPMI-0.05% Tyloxapol and we calculated the volume needed to have a multiplicity of infection (MOI) of 1, using the following conversion: OD600 0.1 = 1x10^7^ CFU/ml. THP1 cells (ATCC® TIB-202 ™) were maintained in complete RPMI1640 (RPMI 1640 HEPES modification, 2 mM L-glutamine, 1 mM sodium pyruvate, 10% fetal bovine serum) and incubated at 37°C with 5% CO2. THP1 phagocytes (2x10^5^ cell/mL) were infected for 4 h in a roller bottle with a MOI of 1 in RPMI-20nM PMA and extracellular bacteria were discarded by washing 5 times in complete RPMI (5 x 402g, 5 min). We dipensed 50 μL/well (10,000 cells/well) of infected THP1 cells in white 384-well plates with 250nl/ well of compound in DMSO.Plates were incubated for 5 days at 37°C/ 5% CO2. Then, 25μl of reconstituted Bright-Glo™ Luciferase Assay System (Promega) were added to each well and plates were incubated at RT for 30 minutes. Finally, the luminescence was read in an Envision system (Perkin Elmer) using these settings: US LUM 384 (cps) 7000004/ Measurement height 0 mm/ Measurement time 0.1 s. Aperture: 384 Plate US Luminescence aperture.

### HepG2 cytotoxicity assay

Actively growing HepG2 cells were removed from a T-175 TC flask using 5 mL Eagle’s MEM (containing 10% FBS, 1% NEAA, 1% penicillin/streptomycin) and dispersed in the medium by repeated pipetting. Seeding density was checked to ensure that new monolayers were not >50% confluent at the time of harvesting. Cell suspension was added to 500 mL of the same medium at a final density of 1.2x10^5^ cells.mL^-1^. This cell suspension (25 μL, typically 3000 cells per well) was dispensed into the wells of 384-well clear-bottom plates (Greiner, cat. # 781091) using a Multidrop instrument. Prior to addition of the cell suspension, the screening compounds (250 nL) were dispensed into the plates with an Echo 555 instrument. Plates were allowed to incubate at 37°C at 80% relative humidity for 48 h under 5% CO_2_. After the incubation period, the plates were allowed to equilibrate at room temperature for 30 min before proceeding to develop the luminescent signal. The signal developer, CellTiter-Glo (Promega) was equilibrated at room temperature for 30 min and added to the plates (25 μL per well) using a Multidrop. The plates were left for 10 min at room temperature for stabilization and were subsequently read using a ViewLux instrument (PerkinElmer).

The human biological samples were sourced ethically and their research use was in accord with the terms of the informed consents.

### Physicochemical properties

#### CLND solubility assay

GSK in-house kinetic solubility assay: 5 μL of 10mM DMSO stock solution diluted to 100 uL with pH7.4 phosphate buffered saline, equilibrated for 1 hour at room temperature, filtered through Millipore Multiscreen HTS-PCF filter plates (MSSL BPC). The filtrate is quantified by suitably calibrated flow injection Chemi-Luminescent Nitrogen Detection [5]. The standard error of the CLND solubility determination is ±30 μM, the upper limit of the solubility is 500 μM when working from 10 mM DMSO stock solution.

#### ChromlogD assay

The Chromatographic Hydrophobicity Index (CHI) [6] values were measured using a reversed phase HPLC column (50 x 2 mm x 3 μM Gemini NX C18, Phenomenex, UK) with fast acetonitrile gradient at starting mobile phase of 100% pH = 7.4 buffer. CHI values are derived directly from the gradient retention times by using a calibration line obtained for standard compounds. The CHI value approximates to the volume % organic concentration when the compound elutes. CHI is linearly transformed into ChromlogD [7] by the formula: ChromlogD = 0.0857CHI-2.00. The average error of the assay is ±3 CHI unit or ±0.25 ChromlogD.

### Exploring the 2D chemogenomics space

We applied a multi-category Naïve Bayesian classifier (MCNBC) that was built and trained using 2D structural and experimental bioactivity information from the ChEMBL database version 16 [8]. In brief, the classifier learns the various categories (in this case protein targets) by considering the enrichment of certain 2D sub-structural features of active compounds across the protein targets. Given a new, unseen compound, the model calculates a Bayesian probability score for each target based on the compound’s individual features and produces a ranked list of likely targets. The model was built in Accelrys Pipeline Pilot (version 8.5) using standard ECFP_6 fingerprints [9] to encode the chemical structures. Further information on the model generation and validation can be found in our previous publication [10]. The statistical significance of the probability scores was assessed with Z-scores. These were computed by calculating the background probability score distribution for each protein target using all the compounds in ChEMBL. Lastly, given that the majority of bioactivities in the ChEMBL database are against human, mouse and rat protein targets, the predicted targets were mapped to their orthologous *M*. *tuberculosis* ones using the OrthoMCL [11] database [12].

### Exploring the 3D structural space

A network of 3D structural similarities between compounds and targets was built to identify the most likely targets of a given compound in the GSK dataset. To explore the structural space, we used nAnnolyze, an improved version of our previously published AnnoLyze algorithm [13], which was based on homology detection through structural superimposition of targets and their interaction networks to small compounds, similar to previously published approaches [14, 15]. Briefly, the new algorithm relies in four pre-built layers of interconnected networks. First, the “GSK Ligand” network where nodes are GSK compounds and edges correspond to their similarity as measured by a previously developed Random Forest Classifier (RFS) score [10]. The RFS classifier predicts whether two small molecules are likely to bind the same target-binding site by comparing their structural and chemical properties. Second, the “PDB Ligand” network where nodes are clusters of highly similar ligands of the Protein Data Bank (PDB) [16] and edges correspond to their similarity measured by the RFS. The “GSK Ligand” network is linked to the “PDB ligand” network by edges corresponding to the compound similarity measure by the RFS. Third, the “PDB Protein” network with nodes corresponding to clusters of highly similar small molecule binding-site of proteins in PDB and edges correspond to their structural similarity as measured with the ProBiS structural superimposition method [17]. Fourth, the previously built “*M*. *tuberculosis* Models” network [10] with nodes corresponding to predicted small molecules binding-sites in three-dimensional models of *M*. *tuberculosis* targets and edges correspond to their structural similarity after comparison by the ProBiS program. The two central networks (that is, “PDB Ligand” and “PDB Protein Binding-Sites” networks) are connected by co-appearance of the compound and the protein in any solved structure in the PDB. The “PDB Protein” and the “*M*. *tuberculosis* Models” networks are linked by the structural comparison between any binding-site in the PDB network and all binding-sites in models from *M*. *tuberculosis*. Finally, once all the networks are constructed, we identified the closest path between any GSK compounds and *M*. *tuberculosis* targets. To score the hit, we used the inverse of the edges weight of the pathway. Next, the final score is normalized to 1 (being 1 the best score and 0 the worst one) and Z-scored. Specifically, two different Z-scores are calculated for each prediction. The first, called Global Z-score, is obtained by running the predictions of all drugs present in DrugBank against all targets and using the global mean and the global standard deviation to Z-score specific predicted pair. The Global Z-score represents how good a prediction is given its score in the constructed network. The second, called Local Z-score, is calculated by running the predictions of all drugs present in DrugBank and retrieving the mean and the standard deviation of the score for a specific target binding-site. The Local Z-score represent how good a prediction is for a specific binding-site; highly promiscuous binding-sites tend to have bad local Z-scores. The nAnnolyze approach was recently evaluated using all the FDA approved drugs present in PDB. In such dataset, the nAnnolyze predictions result in an area under the Receiver Operating Characteristic curve (AUC) of 0.70 [18].

The final entire network of comparisons included the 50 compounds from the GSK dataset, 7,609 unique ligands from the PDB, 28,299 unique compound binding-sites in protein structures from the PDB, and a total of 5,008 structure models from *M*. *tuberculosis*.

### Exploring historical assay data

GSK proprietary compound screening databases were queried for any historical assay data associated with *M*. *tuberculosis* H37Rv (*M*. *tuberculosis* H37Rv) active compounds. The majority of these screens were against human protein targets. The threshold above which compound efficacy against specific human targets was considered significant was defined as pIC_50_ ≥ 5.0 for inhibition or antagonist assays, pEC_50_≥ 5.0 for agonist, activation or modulator assays (*i*.*e*. overall pXC_50_ ≥ 5.0).

Using BLASTP [19] we queried the protein complement of published *M*. *tuberculosis* H37Rv for all human targets accepting a homology cutoff of an E-value ≤1.0e-10 and visual inspection of the alignments. Putative homologous relationships were confirmed by reciprocal BLASTP searches of identified *M*. *tuberculosis* H37Rv homologues against the human RefSeq protein databases (April 2014).

### Statistical assessment of predicted links between compounds and targets

We measured two different statistics to assess the significance of a particular link between a chemical compound and a target pathway. Firstly, we calculated the LogOdds (that is, the odds of an observation given its probability). A feature *i* (in our case, a compound in or a pathway) has a probability (*p*_*i*,*c*_) in the entire dataset and a probability (*p*_*i*,*r*_) of being at the subset of selected compounds/pathways. Their LogOdds are defined as the logarithm of its Odds (*O*_*i*_):

$$O_{i}=\frac{\frac{p_{i,c}}{\left(1-p_{i,c\;}\right)}}{\frac{p_{i,r}}{\left(1-p_{i,r}\right)}}$$

Therefore, Odds higher than 1 (or positive LogOdds) indicate over-occurrence of the compound/pathway in the selected subset. Odds smaller than 1 (or negative LogOdds) indicate under-representation of the compound/pathway in the selected subset. Secondly, a *p-value* score was calculated for each predicted link between a compound and a target pathway using a Fisher's exact test for 2×2 contingency tables comparing two groups of annotations (*i*.*e*., the group of compounds in a given pathway and the group of compounds in the entire dataset) [20]

## Results

### Screening process and drug like properties of hits identified

GSK DDW has very recently added to its Corporate small molecule repository some structurally new 254,000 compounds, known as the “Top-up” library, whose diverse profile reflects the latest intelligence on how specific physicochemical property descriptors (sp^3^ character, lipophilicity/ water solubility, molecular size *etc*.) can affect attrition at the different stages of the drug discovery phase. Given the differentiated structural profile of this compound library, we would expect novel hits that engage new targets beyond those identified during our previous studies [2].

**Fig 1:**
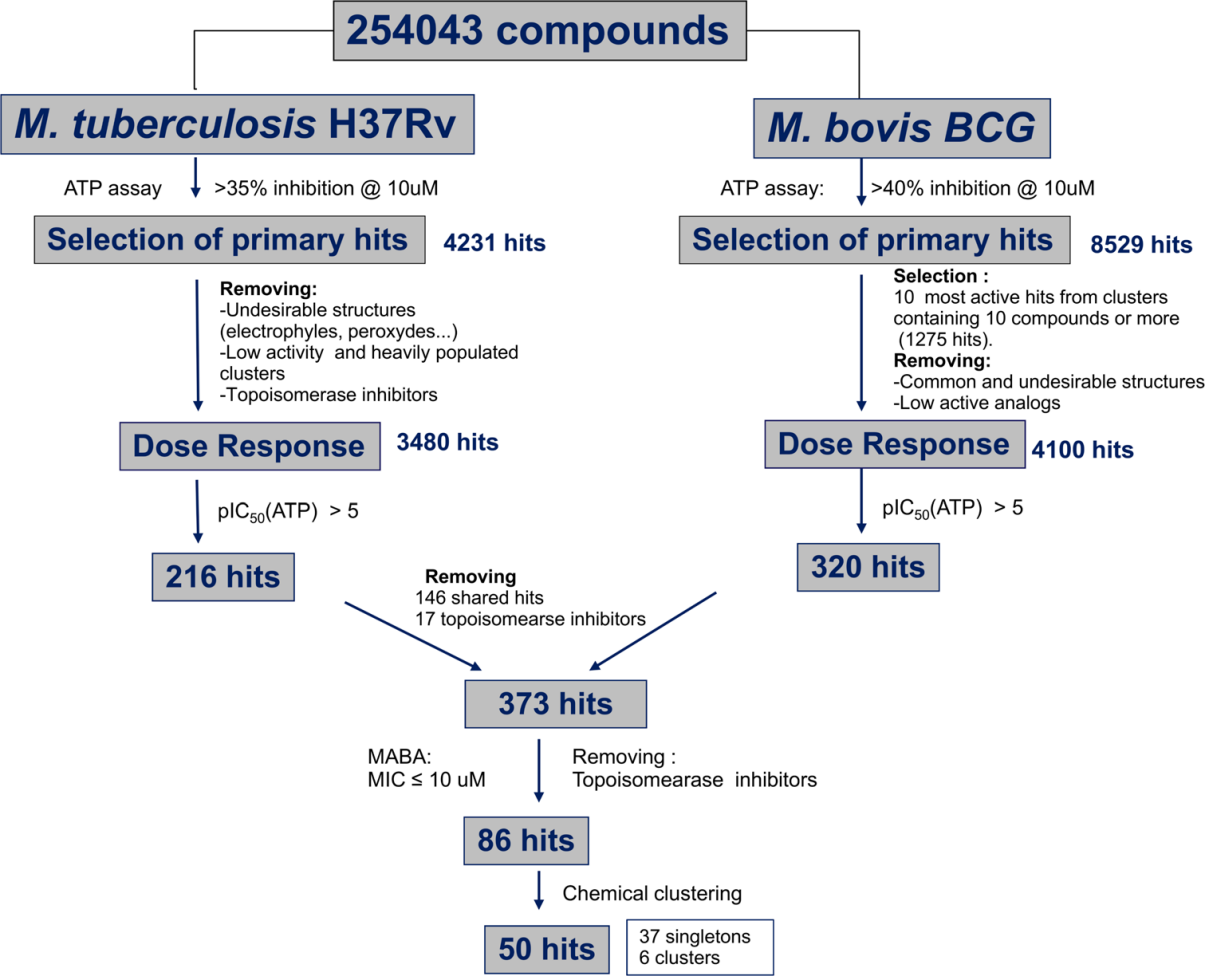
HTS progression cascade leading to 50 confirmed H37Rv-positive compounds.

We have reported previously how non pathogenic and Biosafety Level 2 friendly *M*. *bovis* BCG can act as a modest surrogate to predict the antitubercular activity against *M*. *tuberculosis* H37Rv [2]. It is for this reason that, in this occasion, we decided to undertake parallel screening activities against both strains. Active compounds meeting the pre-established (Fig 1) threshold of activity in the primary ATP antimycobacterial assay, were progressed to evaluation in a resazurin based H37Rv assay. This resulted in the identification of 4,231 compounds that exerted an inhibition of *M*. *tuberculosis* H37Rv growth superior to 35% and, in the case of BCG, 8,529 compounds with growth inhibition values above 40%. At this stage a set of automatic filters directed towards the identification and elimination of a few remaining undesirable structural features such as electrophiles, peroxides and Michael acceptors, was applied resulting in a first reduction on the number hit structures; this set of filters is an updated version of a previously published one.^32^ The fraction of undesirable compounds in the “Top up” library is very small (estimated in 0.08%); however, most of them (ca. 200) showed up in the initial list of hits as they are reactive compounds prone to cytotoxicity effects.

**Fig 2:**
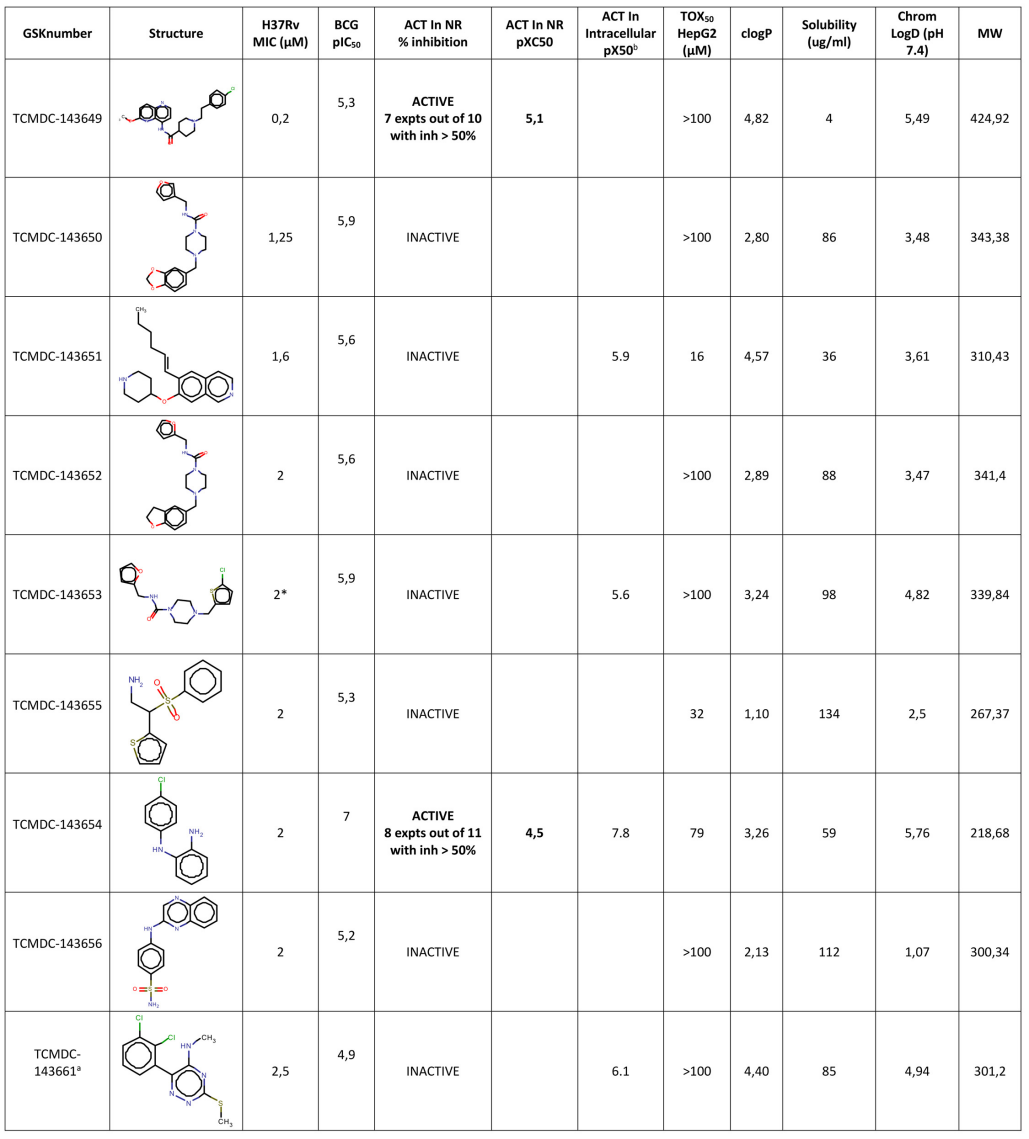
Complete biological profile of selected hit compounds and corresponding physico chemical properties. ^a^ Mtb specific. *This compound has been evaluated against a clinical isolate of *M*.*tuberculosis* resistant to isoniazid and its MIC was in the range of H37Rv (1.6 uM). ^b^ Compounds being tested in the intracellular assay, data will be available from Dryad Digital Repository: http://dx.doi.org/10.5061/dryad.8r351.

This first selection was further narrowed through the application of a specifically designed in house algorithm that helped prioritize highly active structural clusters and remove analogs showing lower percentages of inhibition. Finally, all known antitubercular classes were manually removed. The resulting 320 (BCG screening stream) and 216 (H37Rv screening stream) hits were checked for structural duplication, resulting in 373 compounds that were progressed to Minimum Inhibitory Concentration (MIC) determination against H37Rv in the MABA resazurin assay. In order to progress only the most promising hits, we applied very strict selection criteria: we did not select any compound which either did not reach, at least, the 90% inhibition cutoff or required more than 10μM to reach this threshold. Therefore, only 86 of these 373 compounds were moved forward (MICs lower or equal to 10 μM). This relatively low percentage of actives in the H37Rv MABA assay highlights a lack of correlation between the two readouts employed in this screening effort. The corresponding 86 compounds were then clustered in chemotypes resulting in a final hit list of 50 representative highly potent compounds, including 37 singletons, five clusters of two representatives and one cluster of three representatives (Figs 1 and 2 and Table B in S1 File). When tested for MIC determination in the H37Rv MABA assay, these 50 compounds showed MIC values between 0.2 and 10 μM. Amongst those 50 hits, 7 were Mtb specific and the rest were all identified as hits in both screening campaigns (BCG and Mtb). Activity against *M*. *bovis* BCG (pIC50s) is described in Fig 2 and Table B in S1 File. In order to determine the therapeutic window of the hits, the HepG2 cytotoxicity of each hit was evaluated. From the dose–response results, 24 compounds displayed TOX_50_ between 10 and 100 μM and 26 had no detectable cytotoxic effects (TOX_50_ ≥ 100 μM). The library used in these screens is composed of lead-like compounds with very low lipophilicity. In addition, the few remaining compounds with reactive substructures were automatically removed (see above), as well as topoisomerase inhibitors that inhibit also eucariotic cells (Fig 1). As a result, the final 50 compounds have a very high probability of targetting *Mycobacterium* through specific mechanisms that would explain the low cytotoxicity observed in HepG2 cells.

All the 50 hits were also tested for their activity against non-replicating *M*. *tuberculosis* as described previously [21]. Interestingly, 5 of them were active with pIC_50_ ranging from 4.5 to 5.2 (equivalent to IC_50_´s between 31 and 6 μM). Finally, the inhibition of the intracellular growth of mycobacterium tuberculosis was determined. Out of the 26 representative compounds tested, all but one (TCMDC-143682) were active, with pIC50s above 5. Interestingly, a set of 10 compounds was also tested against clinical isolates resistant to isoniazid (inh^R^) or rifampicin (rif^R^) and all the compounds were as active as against the reference strain H37Rv (Fig 2 and Table C).

**Fig 3:**
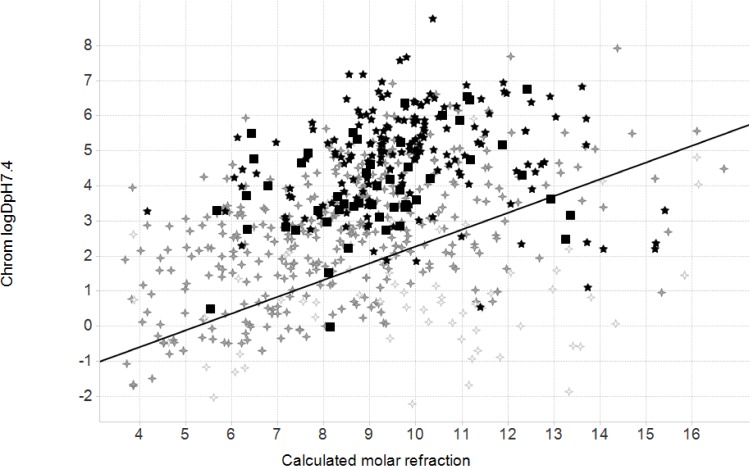
Plot of calculated chromatographic logD_7.4_ versus calculated molar refraction (CMR). All data were generated using the latest version of the GSK calculator. Grey crosses represent marketed drugs with >30% oral bioavailability, white crosses <30% oral bioavailability, and the two disclosed sets by black squares (the current 50 compounds) or black stars (the CMC2013 set of 177). The line represents a discriminator between likely good and bad permeability. The chromatographic logD scale gives values approximately two units higher than the traditional distribution values assessed in octanol/water.

This new compound set again resides comfortably within the range of properties occupied by marketed drugs and on average has a slightly lower lipophilicity than the first set (see Figs 2 and 3; Table B in S1 File and Figures C-E in S1 File). The compounds identified generally presented a combination of a reasonable level of solubility and anti-mycobacterial activity, indicating their attractiveness as starting points for lead optimisation. No statistically significant difference in the distributions of physicochemical properties was observed between the 7 H37Rv-specific compounds and rest of the compounds, although they are structurally dissimilar.

### Target Prediction

The final 50 compounds were computationally analyzed with the goal of identifying their likely target proteins. Our computational approach integrates 2D chemogenomics space (CHEM), structural comparisons (STR) and historical bioassay data (HIST). The results from this analysis were also compared to those from our previous analysis [10].

#### 2D Chemogenomics space (CHEM)

The exploration of the chemical space allowed us to identify likely targets (Table 1) for the input compounds based on their structural similarity to compounds with experimentally validated targets deposited in the ChEMBL database [8]. We applied a multi-category naïve Bayesian classifier (MCNBC) that was built and trained using structural and bioactivity information from the ChEMBL database [8]. Given a new compound, the model calculates a likelihood score based on the molecule's individual sub-structural/fingerprint features and produces a ranked list of likely targets.

**Table 1:** Significant links between compound families and targets.

Compound	FamID	Target	Pathway	Essentiality Prediction
**TCMDC-143652**	1	Rv3569c	Degradation of aromatic compounds (mtu01220) Steroid degradation (mtu00984)	Non
**TCMDC-143653**	1	Rv3569c	Degradation of aromatic compounds (mtu01220) Steroid degradation (mtu00984)	Non
**TCMDC-143657**	1	Rv3569c	Degradation of aromatic compounds (mtu01220) Steroid degradation (mtu00984)	Non
**TCMDC-143650**	1	Rv3569c	Degradation of aromatic compounds (mtu01220) Steroid degradation (mtu00984)	Non
**TCMDC-143666**	3	Rv2855	Glutathione metabolism (mtu00480)	Yes
**TCMDC-143687**	3	Rv0427c	Base excision repair (mtu03410)	Non
	3	Rv1629	Base excision repair (mtu03410)	Yes
	3	Rv2855	Glutathione metabolism (mtu00480)	Yes
**TCMDC-143688**	5	Rv1284	Nitrogen metabolism (mtu00910)	Yes
**TCMDC-143670**	5	Rv3273	Nitrogen metabolism (mtu00910)	Non
	5	Rv3588c	Nitrogen metabolism (mtu00910)	Non
	5	Rv1284	Nitrogen metabolism (mtu00910)	Yes
	5	Rv3273	Nitrogen metabolism (mtu00910)	Non
	5	Rv3588c	Nitrogen metabolism (mtu00910)	Non
**TCMDC-143649**	9	Rv0194	ABC transporters (mtu02010)	Non
**TCMDC-143690**	13	Rv1284	Nitrogen metabolism (mtu00910)	Yes
	13	Rv3588c	Nitrogen metabolism (mtu00910)	Non
**TCMDC-143655**	29	Rv1151c	Amino sugar and nucleotide sugar metabolism (mtu00520)	Non
**TCMDC-143686**	36	Rv0233	Purine metabolism (mtu00230)	Non
	36	Rv0733	Purine metabolism (mtu00230)	Non data
	36	Rv1843c	Purine metabolism (mtu00230)	Non
	36	Rv2584c	Purine metabolism (mtu00230)	Non
	36	Rv3275c	Purine metabolism (mtu00230)	Yes
	36	Rv3307	Purine metabolism (mtu00230)	Non
	36	Rv3411c	Purine metabolism (mtu00230)	Yes
**TCMDC-143685**	38	Rv1905c	D-Arginine and D-ornithine metabolism (mtu00472) Penicillin and cephalosporin biosynthesis (mtu00311)	Non

In total, the 50 compounds resulted in 262 statistically significant target associations (at a Z-score > 2.0) to 221 different proteins in the ChEMBL database from 24 different organisms (57% of hits are to human proteins). A simple orthology search the OrthoMCL database against the *M*. *tuberculosis* proteins from this set resulted in 128 compound-target relationships for 61 *M*. *tuberculosis* proteins, with detectable orthology to 16 organisms (Table C in S1 File).

#### Historical assay space (HIST)

We used the historical GSK bioassay data to develop hypotheses for the anti-mycobacterial mode of action for the active compounds. Using conservative activity thresholds (pXC50 ≥ 5.0) we found that among the 50 compounds active against *M*. *tuberculosis* H37Rv, 25 displayed additional activity in 65 different historical biochemical assays against human (50 unique genes), bacterial (1 gene) and viral (1 gene) putative targets (Figure A.A in S1 File). Some compounds were present in multiple historical assays resulting in a total of 93 assay experiments (Figure A.B in S1 File).

The largest human target classes were G protein coupled receptors (GPCRs) and protein kinases, which might partly reflect the relative abundance of different ligand classes in GSK’s pharmacological screening collection. We searched for orthologous sequences of the human assayed proteins in the *M*. *tuberculosis* H37Rv genome using conservative criteria (BLASTP E-value ≤1.0e-10) for assigning human-*Mycobacterium* protein homology. Although there are significant evolutionary differences between *Mycobacterium* and human genomes in terms of both gene content and amino acid sequence divergence, we still found 17 *M*. *tuberculosis* H37Rv gene homologues (Table A in S1 File), which fell into different target class categories (Figure A in S1 File), including kinases (8 genes), cytochromes (6 genes), other enzymes (2 genes) and ion channels (2 genes).

The specific predictions from the historical assay space search are detailed in S1 File.

#### 3D Structural space (STR)

Finally, we applied a Random Forest Score that identified structural similarities between any compound in the dataset and ligands from the PDB [22]. Each compound in the *M*. *tuberculosis* H37Rv dataset is compared to ~7,600 ligands for which there are known complex structures in the PDB, identifying structural similarities to be included in a pre-built network of structural relationships between ligands and targets. In total, the 50 compounds resulted in 1,890 significant target associations (global Z-score < -1) to proteins in a set of modeled three-dimensional structures from the *M*. *tuberculosis* proteome (data not shown).

#### Predicted targets

The similarities and differences of the predictions of the three independent approches are detailed in S1 File.

**Fig 4:**
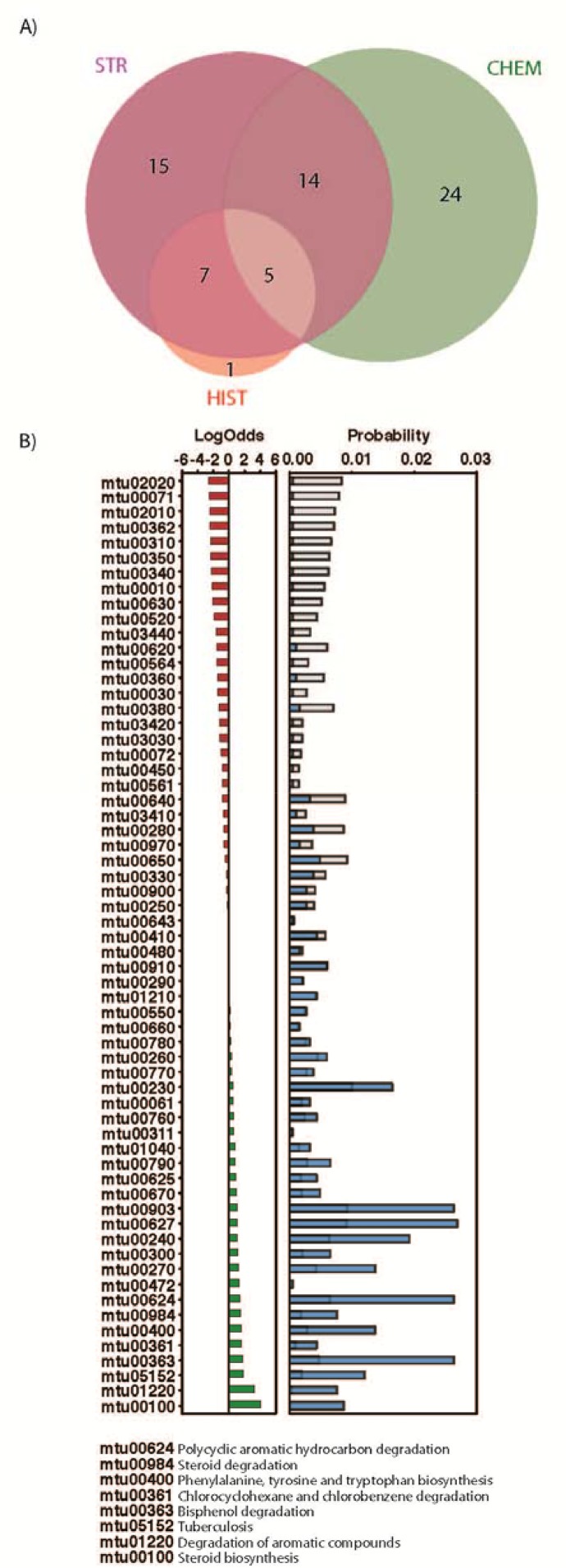
Predicted KEGG pathways targeted by the GSK compounds. A) Venn diagram with common pathways from the three different approaches. B) Most under and over-represented pathways in our predictions. Panels A) and B) with the same representation as in Figure E in S1 File.

There were a total of 1,044 unique *M*. *tuberculosis* targets associated with 112 pathways annotated in the KEGG database [23]. The KEGG being a suite of databases and associated software for understanding and simulating higher-order functional behaviours of the cell or the organism from its genome information. The “mtu” identifiers below refer to the relevant KEGG pathway ids. The three orthogonal approaches identified 66 different pathways (Fig 4A) associated to the 50 hit compounds. The relative increment in the number of putatively affected pathways per compound comparing to the previous TCAMS-TB dataset [10] can be explained by the higher structural diversity of the novel top-up library. Within the commonly identified pathways, there are many associated with amino acid and nucleotide metabolism, e.g. the mtu00260 (Glycine, serine and threonine metabolism), mtu00380 (Tryptophan metabolism), mtu00330 (Arginine and proline metabolism), mtu00270 (Cysteine and methionine metabolism), mtu00240 (Pyrimidine metabolism), mtu00230 (Purine metabolism), mtu00360 (Phenylalanine metabolism), mtu00290 (Valine, leucine and isoleucine biosynthesis). Some of them also appear overrepresented in the predictions, e.g phenylalanine, tyrosine and tryptophan biosynthesis (Fig 3B). Interestingly, there are others overrepresented pathways not directly associated with amino acid metabolism such as mtu05152, mtu01220 (Degradation of aromatic compounds), or mtu00363 (Bisphenol degradation).

To assess the significance of the compound-target predictions using the three different approaches, we calculated a t-statistics p-value of any compound family-KEGG pathway link (Methods). There are 8 compounds families significantly associated (p-value < 0.005) to 10 different KEGG pathways. The threshold used in this study is less restrictive than in the prior study [10] due to the smaller number of compounds. This results in a higher number of associations found between compounds and KEGG pathways. Family 1 is significantly linked with both mtu01220 (Degradation of aromatic compounds) and mtu00984 (Steroid degradation). Specifically, the link found by compounds TCMDC-143652, TCMDC-143653, TCMDC-143657, and TCMDC-143650 targeting Rv3569c (4,9-DHSA Hydrolase) involved both pathways. Family 3 is significantly associated with two different KEGG pathways, mtu00480 (Glutathione metabolism) and mtu003410 (Base excision repair). Specifically, compounds TCMDC-143687 and TCMDC-143666 are predicted to hit Rv2855 (NADPH-dependent mycothiol reductase) involved in Glutathionine metabolism and essential for the survival of the bacteria [24], while TCMDC-143687 is predicted to hit the base excision repair pathway through Rv0427c (Exodeoxyribonuclease III protein XthA) and Rv1629 (DNA polymerase I PolA) being the later essential for the growth of the bacteria [24, 25]. Family 5 has a strong association (p-value 1.0e-08) with mtu00910 (Nitrogen metabolism pathway) an essential pathway for the bacteria survival. Specifically, compounds TCMDC-143688 and TCMDC-143670 are predicted to hit Rv1284 (beta-carbonic anhydrase), Rv3273 (carbonate dehydratase) and Rv3588c (beta-carbonic anhydrase CanB) three proteins involved in the Nitrogen metabolism, where Rv1284 play a key role in the essentiality of this pathway. Moreover, compound TCMDC-143690 belonging to singleton family 13, is also predicted to interact with Rv1284 and Rv3588c targeting the nitrogen metabolism pathway with a completely different chemical scaffold. Another interesting significant link is the compound TCMDC-143648 targeting the mtu02010 (ABC transporters pathway) through the Rv0194 target (transmembrane multidrug efflux pump). Family 29 composed by compound TCMDC-143655 is predicted to interact with Rv1151c (transcriptional regulatory protein), which is involved in transcriptional mechanism and belongs to mtu00520 (amino sugar and nucleotide sugar metabolism). Family 36 with compound TCMDC-143686 is significantly associated with the mtu00230 (purine metabolism) pathway. This compound is predicted to attack the pathway by targeting 7 different proteins in this pathway (Rv0233, Rv0733, Rv1843c, Rv2584, Rv3275c, Rv3307, and Rv3411c). Among the predicted targets, there are two essential for the bacterial survival [24, 25], the N5-carboxyaminoimidazole ribonucleotide mutase (Rv3275c) and the inosine-5'-monophosphate dehydrogenase (Rv3411c). Finally, a significant link between Family 38 (TCMDC-143685) and pathways mtu00472 (D-arginine and D-ornithine metabolism) and mtu00311 (Penicillin and cephalosporin biosynthesis) was also observed trough the target Rv1905c (a Probable D-amino acid oxidase Aao).

## Discussion

Screening for new antitubercular inhibitors in whole cell based assays still sustains a high proportion of the drug discovery pipeline against TB. While this choice of screening strategy is not devoid of its own specific issues [26] the completion of a number of screening efforts and, most importantly, the public release of these datasets, is enabling the in depth validation of novel Mode-of-Actions (MoA) against TB [27–32]. This target elucidation work, in time, is promising to open up new opportunities for TB drug discovery where the limitations associated with the medicinal chemistry optimisation of hits identified by whole cell screening can be addressed through the support provided by technologies typically associated with target based discovery programs, e.g. particular target assays and crystallography. We expect that by accessing these technologies, a more rational understanding of the optimization process and the early identification of potential target related toxicological liabilities could be attained.

It is with this goal in mind that we here present a novel set of antitubercular compounds together with some developability parameters that should provide the TB R&D community with novel chemical starting points for further discovery or, more importantly, future target identification programs. The present release incorporates compounds which, on average, would appear to be in more favourable physical space than those in the previous publication [2, 10]. Given the predominantly intracellular lifestyle of Mtb and the suspected impact of non-replicating bacteria in TB chemotherapy [33, 34], we decided to investigate whether the compounds were capable of inhibiting Mtb growth in THP-1 cells and were active against non-replicating bacteria. 96% of the compounds tested in the intracellular assay were found to be active and 10% of the whole set retained activity in the non-replicating assay. On the basis of the drug-like properties presented in Fig 2 and Table B in S1 File, ten molecules were selected for further characterisation against isoniazid and rifampicin clinical resistant isolates. All compounds were found to be active within the same range as the reference strain H37Rv.

Interestingly, 7 compounds of the set were Mtb specific (inactive against M. bovis BCG). While a number of especulative explanations can be postulated, e.g. differences in permeability, active transport, metabolic state, etc., this lack of correlation highlights the risks associated with the use of non pathogenic surrogate strains in antitubercular research.

To further characterize the activity of the novel antitubercular compounds, we have integrated a series of orthogonal computational approaches for predicting their putative targets. Our analysis found nine chemical families targeting 21 different proteins from 13 biochemical pathways in *M*. *tuberculosis*. Within the 21 proteins, there are 5 assessed as essential in previous studies. The essentiality of these targets makes them top priority targets for further validation. However, some non-essential targets can have a key role in TB infection in-vivo and therefore we should consider them in the search of new strategies for defeating TB. Our target identification work aims to facilitate further chemical and biochemical experiments to optimize the properties of the compounds against TB. Optimally, additional computational approaches could then interrogate the newly generated compounds to further characterize their mode-of-action. This iterative process is very desirable to maximize the impact of the openly released new compounds against TB. In particular, we also release the 3D structural models for the significant predictions of targets and compounds identified by the STR approach. Such models and the predicting binding site could be used for computational docking or molecular dynamics analysis to further validate our prediction

## Supporting Information

S1 File Supporting information, Figures and Tables. **Figure A. Target class space.** A) For positive hits in *M*. *tuberculosis* H37Rv screens, the distribution of human target classes affected by compounds based on known human protein potency and selectivity criteria as described in the text. The number of human targets is indicated for each class as well as the potential number of *Mtb* homologous genes (in parentheses). B) Distribution of 25 compounds screened against 1 or more targets having pIC50 or pEC50 values > 5.5 in 65 assays by human target classes. Some compounds have historical assay information and potency against multiple target classes. Also indicated is the number of assays against targets with putative homologues in *M*. *tuberculosis* (in parentheses). **Figure B. Box plot of average PFI** (calculated Chrom Log D7.4 + #Ar) distribution of the 177 compounds released previously [2], the current 50 hits and a representative set of oral drugs. **Figure C**. **Box plot of average calculated Chrom Log D7.4** distribution of the 177 compounds released previously [2], the current 50 hits and a representative set of oral drugs. **Figure D. Box plot of average calculated molar refraction** (CMR) distribution of the 177 compounds released previously [2], the current 50 hits and a representative set of oral drugs. **Figure E. Subset of GSK compounds with predicted targets. A**) Venn diagram with common compounds with predictions from the three different approaches (that is, in green from the search of the chemogenomics space, in purple from the search of the structural space, and in red from the historical data). **B**) Venn diagram with common compound families with predictions from the three different approaches. **C**) Most under and over-represented chemical families in our predictions. Upper plot shows the probability of finding a given family in the original dataset (grey bars) compared to the probability of finding it in the dataset with predicted targets (blue bars). Lower plot shows the log odds per selected family (*i*.*e*., absolute log odds larger than 0.5). **Table A. Predicted *Mtb*H37Rv gene targets** based on homology to 65 historical human target assays for 25 compounds. Notes: ^a^ Human target classes are defined in the text. Some compounds were reported active across more than one target class hence the greater number of total than tested compounds. ^b^ *M*. *tuberculosis* H37Rv homologs determined by BLASTP searches using human target proteins [19]. ^c^ Essentiality scoring based on Sassetti et al.[24]. NE = No Evidence from these sources. **Table B.** Complete biological profile of selected hit compounds and corresponding physico chemical properties. **Table C.** Target association based on the structural similarity of the hits to compounds with experimentally validated targets deposited in the ChEMBL database.
(PDF)
