# Supplementary material for: Release of 50 new, drug-like compounds and their computational target predictions for open source anti-tubercular drug discovery
Source: PLoS One. 2015 Dec 7;10(12):e0142293. doi: 10.1371/journal.pone.0142293 (PMC4671658; doi:10.1371/journal.pone.0142293)
Supplement: S1 File — Figure A. Target class space. A) For positive hits in M. tuberculosis H37Rv screens, the distribution of human target classes affected by compounds based on known human protein potency and selectivity criteria as described in the text. The number of human targets is indicated for each class as well as the potential number of Mtb homologous genes (in parentheses). B) Distribution of 25 compounds screened against 1 or more targets having pIC50 or pEC50 values > 5.5 in 65 assays by human target classes. Some compounds have historical assay information and potency against multiple target classes. Also indicated is the number of assays against targets with putative homologues in M. tuberculosis (in parentheses). Figure B. Box plot of average PFI (calculated Chrom Log D7.4 + #Ar) distribution of the 177 compounds released previously [2], the current 50 hits and a representative set of oral drugs. Figure C. Box plot of average calculated Chrom Log D7.4 distribution of the 177 compounds released previously [2], the current 50 hits and a representative set of oral drugs. Figure D. Box plot of average calculated molar refraction (CMR) distribution of the 177 compounds released previously [2], the current 50 hits and a representative set of oral drugs. Figure E. Subset of GSK compounds with predicted targets. A) Venn diagram with common compounds with predictions from the three different approaches (that is, in green from the search of the chemogenomics space, in purple from the search of the structural space, and in red from the historical data). B) Venn diagram with common compound families with predictions from the three different approaches. C) Most under and over-represented chemical families in our predictions. Upper plot shows the probability of finding a given family in the original dataset (grey bars) compared to the probability of finding it in the dataset with predicted targets (blue bars). Lower plot shows the log odds per selected family (i.e., absolute log odds l [file pone.0142293.s001.pdf]

Supplemental Information for article

Release of 50 new, drug-like compounds and their computational target predictions for open source anti-tubercular drug discovery

María Jose Rebollo-Lopez, Joël Lelièvre*, Daniel Alvarez-Gomez, Julia Castro-Pichel, Francisco Martínez-Jiménez, George Papadatos, Vinod Kumar, Gonzalo Colmenarejo, Grace Mugumbate, Mark Hurle , Vanessa Barroso, Rob J. Young, María Martinez-Hoyos, Rubén González del Río, Robert H. Bates; Eva Maria Lopez-Roman, Alfonso Mendoza-Losana, James R. Brown, Emilio Alvarez-Ruiz, Marc A. Marti-Renom, John P. Overington, Nicholas Cammack, Lluís Ballell & David Barros-Aguire.

* Corresponding author.

Historical assay space (HIST)

As described in Results, the human-Mycobacterium homology exercise for all the proteins flagged in the historical GSK bioassay data identified 17 Mtb H37Rv gene homologues.

Two kinases (PknA and PknB) and one enzyme (ilvE) among our putative human-*Mycobacterium* target homologs were previously reported to be essential (1-3). Mycobacterial kinases are related to metazoan serine/threonine protein kinases and have been previously proposed as potential anti-TB drug targets (4). The gene ilvE encodes for a branched-chain amino-acid transaminase which catalyses methionine formation from ketomethiobutyrate. Venos et al. showed that inhibition of this enzyme by selected aminooxy compounds can inhibit bacterial cell growth *in vitro* (5). Other interesting putative Mtb H37Rv targets determined from human historical assays include Rv1151c, which encodes a sirtuin-like deacetylase that can reactivate acetyl-CoA synthetase via NAD+-dependent deacetylation (6, 7). Rv1151c shares significant homology to mammalian SIRT1 for which a single compound assay was found positive in both the SIRT1 and Mtb screening sets. Two putative Mtb H37Rv genes Rv3728 and Rv3239c identified as homologues to human ion channels are efflux pumps related to drug resistance (8).

Subset of compounds with predicted targets

Of the 50 compounds within the Top Up dataset, seven (TCMDC-143662, TCMDC-143654, TCMDC-143688, TCMDC-143651, TCMDC-143666, TCMDC-143650, TCMDC-143665) gave raise to target predictions in the three independent approaches (Fig E in S1 File. A). As for the GSK dataset (9), the majority of target predictions were obtained by the CHEM approach (34 compounds with predicted targets), followed by the STR approach (29) and the HIST approach

(15). We also noticed an important increment in number of compounds for which targets could be predicted (90% for the Top Up dataset versus the 55% of the previous GSK dataset analyzed (9). The 50 compounds were classified in 41 families, while the 7 compounds hit by the three orthogonal approaches belong to 7 different compound families (Fig E in S1 File. B), which is indicative of the ability of the methods to retrieve predictions for different chemical scaffolds.

To identify whether the three different approaches predicted targets for specific families in the dataset, we calculated the probability (LogOdd) of a given compound family to appear in the list of selected compounds, given their different distributions in the original dataset (Fig E.C). This analysis aimed at identifying possible biases or artifacts specific to each of the three independent methods used in our integrative approach. Eight compound families were over-represented (LogOdds > 0.5) in the Top Up dataset while 20 families were underrepresented (LogOdds < -0.5) in the dataset. Interestingly, most of the overrepresented families in the predictions are singletons. The only overrepresented family having more than one member is family_4 (several compounds). The most overrepresented family is family 36 (TCMDC-143686) with a LogOdds of 1.74. This compound is a very promiscuous compound in our predictions with 64 different predicted targets by either the CHEM or the STR approach. Conversely, the family 32 (TCMDC-143696) appears as the most underrepresented (LogOdds = -3.5) with only one prediction by the CHEM approach.

The CHEM approach identified the highest number of different pathways followed by the STR and the HIST. A total of 5 unique pathways were commonly predicted by the three approaches,

while 14 were identified by the STR/CHEM approaches and 7 by the STR/HIST (Fig E.A and Table 1).

Supplemental Figures

Fig A.

Target class space**.** A) For positive hits in *M. tuberculosis* H37Rv screens, the distribution of human target classes affected by compounds based on known human protein potency and selectivity criteria as described in the text. The number of human targets is indicated for each class as well as the potential number of *Mtb* homologous genes (in parentheses). B) Distribution of 25 compounds screened against 1 or more targets having pIC50 or pEC50 values > 5.5 in 65 assays by human target classes. Some compounds have historical assay information and potency against multiple target classes. Also indicated is the number of assays against targets with putative homologues in *M. tuberculosis* (in parentheses).

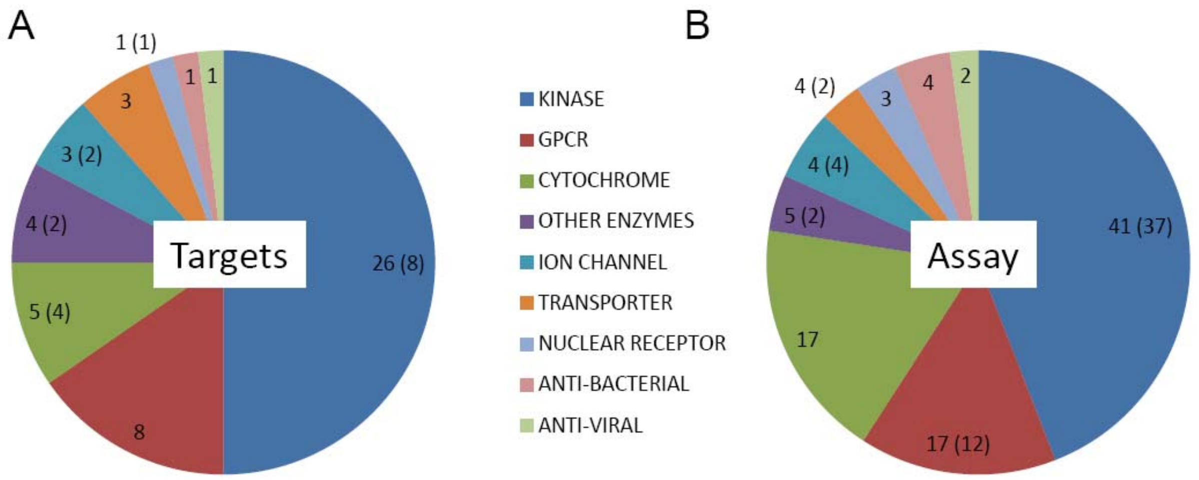

Fig B

Box plot of average PFI (calculated Chrom Log $D_{7.4}$ + #Ar) distribution of the 177 compounds released previously (10), the current 50 hits and a representative set of oral drugs.

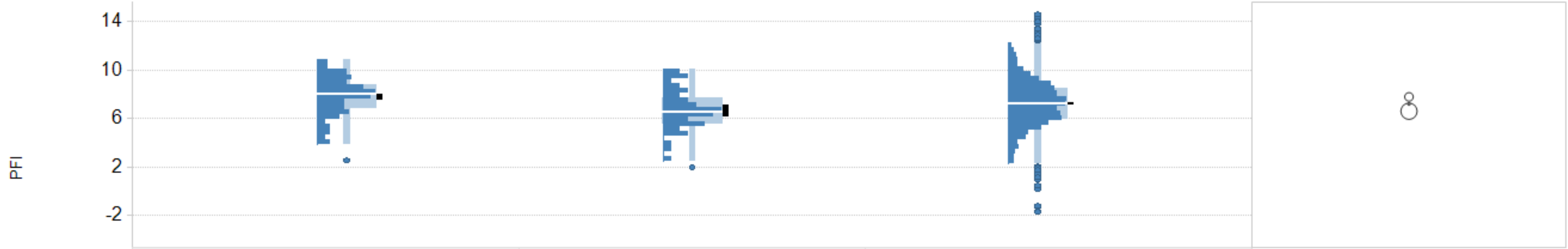

	2013-177	2014-50	oral drugs
Count	177	50	2011
Median	7.96	6.51	7.22
Outliers	1	1	47

Alpha level = 0.05
Root MSE = 2.028
sqrt(2)q* = 3.317

TB sets vs oral

Fig C

Box plot of average calculated Chrom Log $D_{7.4}$ distribution of the 177 compounds released previously (10), the current 50 hits and a representative set of oral drugs.

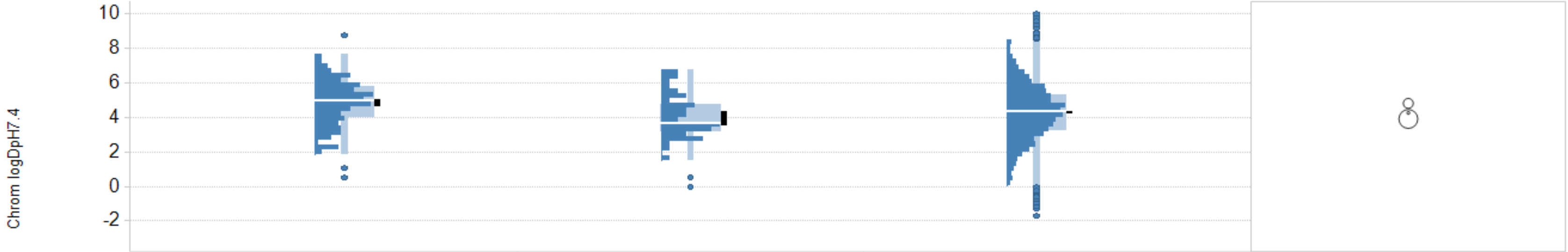

	2013-177	2014-50	oral drugs
Count	177	50	2014
Median	4.98	3.68	4.32
Outliers	3	2	40

Alpha level = 0.05
Root MSE = 1.647
sqrt(2)q* = 3.317

TB sets vs oral

Fig D

Box plot of average calculated molar refraction (CMR) distribution of the 177 compounds released previously (10), the current 50 hits and a representative set of oral drugs.

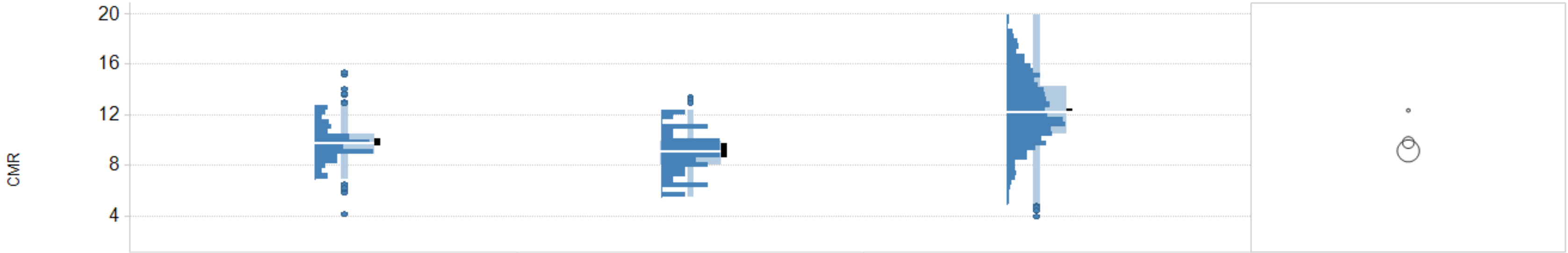

	2013-177	2014-50	oral drugs
Count	177	50	2011
Median	9.7	9.095	12.15
Outliers	19	3	5

Alpha level = 0.05
Root MSE = 2.614
sqrt(2)q* = 3.317

TB sets vs oral

Fig E. Subset of GSK compounds with predicted targets.

A) Venn diagram with common compounds with predictions from the three different approaches (that is, in green from the search of the chemogenomics space, in purple from the search of the structural space, and in red from the historical data). **B**) Venn diagram with common compound families with predictions from the three different approaches. **C**) Most under and over-represented chemical families in our predictions. Upper plot shows the probability of finding a given family in the original dataset (grey bars) compared to the probability of finding it in the dataset with predicted targets (blue bars). Lower plot shows the log odds per selected family (*i.e.*, absolute log odds larger than 0.5).

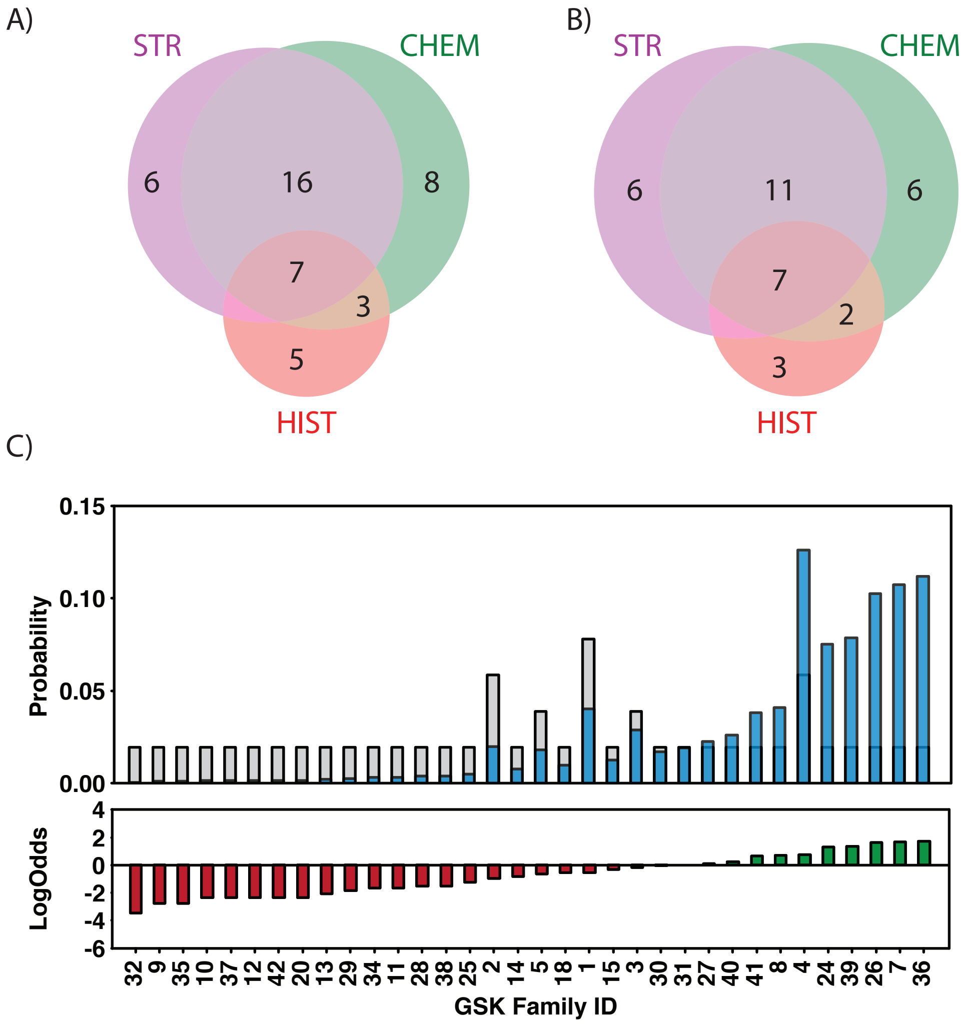
A)
STR
CHEM
HIST
6
16
8
7
3
5
B)
STR
CHEM
HIST
6
11
6
7
2
3
C)
Probability
0.15
0.10
0.05
0.00
LogOdds
4
2
0
-2
-4
-6
32 9 35 10 37 12 42 20 13 29 34 11 28 38 25 2 14 5 18 1 15 3 30 31 27 40 41 8 4 24 39 26 7 36
GSK Family ID

Supplemental Tables

Table A. Predicted *Mtb*H37Rv gene targets based on homology to 65 historical human target assays for 25 compounds.

Human Target Class[a]	No. of Positive Compounds per Target Class	Putative *M. tuberculosis* H37Rv Homolog[b]			Essentiality[c]
		Gene	Product	RefSeq ID	
Kinase	5	pknA	transmembrane serine/threonine-protein kinase A	NP_214529.1	Essential
		pknB	transmembrane serine/threonine-protein kinase B	NP_214528.1	Essential
		pknD	Ser/Thr protein kinase	NP_215446.1	NE
		pknE	Probable transmembrane serine/threonine-protein kinase E PknE	NP_216259.1	NE
		pknH	putative transmembrane serine/threonine-protein kinase H	NP_215782.1	NE
		pknJ	putative transmembrane serine/threonine-protein kinase J	NP_216604.1	NE
		pknK	putative serine/threonine-protein kinase transcriptional regulatory protein K	NP_217596.1	NE
		pknL	putative transmembrane serine/threonine-protein kinase L	NP_216692.1	NE
Enzymes	1	ilvE (Rv2210c)	Branched-chain amino acid transaminase	NP_216726.1	Essential
	1	Rv1151c	Transcriptional regulatory protein	NP_215667.1	NE
Cytochrome	6	cyp132	putative cytochrome P450 132	YP_177807.1	NE
		cyp136	putative cytochrome P450 136	NP_217575.1	NE
		cyp137	Probable cytochrome P450 137 Cyp137	NP_218202.1	NE
		cyp138	Probable cytochrome P450 138 Cyp138	NP_214650.1	NE
Antibacterial	4	gyrB	DNA gyrase subunit B	NP_214519.1	Essential
Ion Channel	2	Rv3728	Probable conserved two-domain membrane protein	NP_218245.1	NE
		Rv3239c	Probable conserved transmembrane transport protein	NP_217756.1	NE
G-PCR	8	No homolog			
Nuclear Receptor	3	No homolog			
Transporter	2	No homolog			
Other Enzymes	3	No homolog			

Other Ion Channel	3	No homolog			
Antiviral	1	No homolog			
Total Compounds	39				

Footnotes

[a] Human target classes are defined in the text. Some compounds were reported active across more than one target class hence the greater number of total than tested compounds.

[b] *M. tuberculosis* H37Rv homologs determined by BLASTP searches using human target proteins (11).

[c] Essentiality scoring based on Sassetti et al.(3). NE = No Evidence from these sources.

Table B. Complete biological profile of selected hit compounds and corresponding physico chemical properties.

GSK number	Structure	H37Rv MIC (µM)	BCG pIC50	ACT In NR % Inhibition	ACT in NR pXC50 MEAN	ACT in Intracellular pXC50 [b]	ACT in resistant strains MIC (µM)	TOX_{50} HepG2 (µM)	clogP	Solubility (ug/ml)	Chrom LogD (pH 7.4)	MW
TCMDC-143660	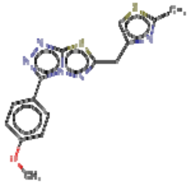	2.5	5.3	INACTIVE			1,3 (inh^R)	>100	2.28	95	2.5	343.43
TCMDC-143659	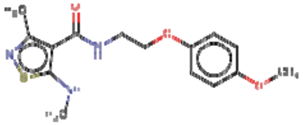	2.5	5.5	INACTIVE			0,9 (inh^R)	>100	2.77	121	2.5	321.4
TCMDC-143657	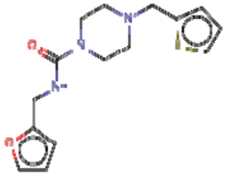	2.5	5.1	INACTIVE		5.4		>100	2.48	105	2.5	305.4
TCMDC-143658	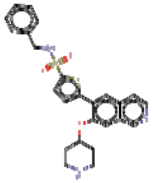	2.5	5.7	INACTIVE		5.4		12.6	3.74	33	2.5	479.61

TCMDC-143662	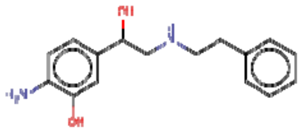	2.9	5.7	INACTIVE			1,3 (inh^{R})	50	1.15	53	2.9	272.34
TCMDC-143663	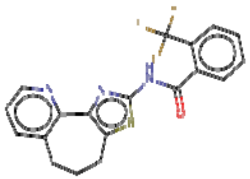	3.7	5.6	INACTIVE		6.2	1,3 (inh^{R})	79	3.56	9	3.7	389.39
TCMDC-143664 [a]	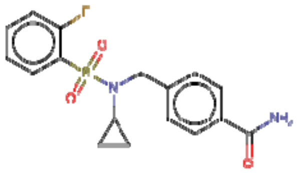	3.75	4.9	INACTIVE		6.7	5 (inh^{R})<br>5 (rif^{R})	>100	2.35	133	3.75	348.39
TCMDC-143665 [a]	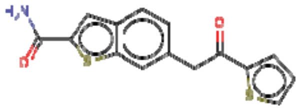	3.8	4.8	INACTIVE				>100	2.84	8	3.8	301.38

TCMDC-143666	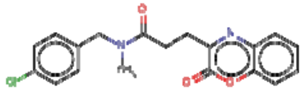	3.9	5.4	ACTIVE 6 expts out of 10 with inh > 50%	**4.9**		1,3 (inh^R)	>100	2.09	127	3.9	356.8
TCMDC-143668	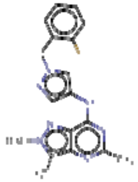	4	5.6	INACTIVE		6		>100	3.40	23	4	351.38
TCMDC-143667	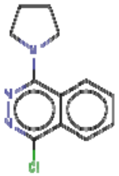	4	5.5	INACTIVE			0,6 (inh^R)	>100	2.47	100	4	233.7
TCMDC-143669	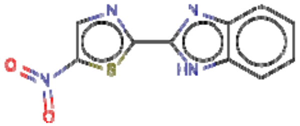	4.4	5.4	INACTIVE				10	2.23	16	4.4	246.24

TCMDC-143670	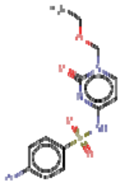	4.7	5.5	INACTIVE			20 (inh^{R}) 1,3 (rif^{R})	>100	-0.16	52	4.7	324.36
TCMDC-143676	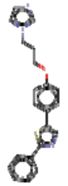	5	5.1	INACTIVE		5.3		25	4.78	3	5	361.46
TCMDC-143674	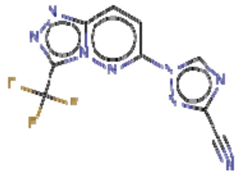	5	5.1	INACTIVE				>100	-0.83	105	5	280.17
TCMDC-143673	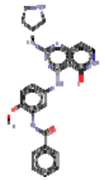	5	5.3	INACTIVE		5.5		25	2.42	106.5	5	471.51

Footnotes

[a] MTB specific

[b] all data will be available from the ChEMBL-NTD data base:

Table C. Target association based on the structural similarity of the hits to compounds with experimentally validated targets deposited in the ChEMBL database.

Compound number	ChEMBL_Pref_Name	ChEMBL_Organism	NB_score	Z_score	TB_Protein	TB_Protein_Name
TCMDC-143669	Methionyl-tRNA synthetase	Staphylococcus aureus subsp. aureus MW2	122,470,017,396,892	295,684,474,590,096	O05593	Methionine--tRNA ligase (EC 6.1.1.10) (Methionyl-tRNA synthetase) (MetRS)
TCMDC-143686	Sphingosine kinase 1	Homo sapiens	816,528,670,876,943	207,379,155,812,207	O05848	Diacylglycerol kinase catalytic domain-containing protein
TCMDC-143698	Pyruvate kinase isozymes M1/M2	Homo sapiens	84,135,551,373,979	217,578,624,971,778	O06134	Pyruvate kinase (PK) (EC 2.7.1.40)
TCMDC-143684	Luciferin 4-monooxygenase	Photuris pennsylvanica	159,655,040,138,576	237,473,084,117,749	O06168	PROBABLE FATTY-ACID-CoA LIGASE FADD35 (FATTY-ACID-CoA SYNTHETASE) (FATTY-ACID-CoA SYNTHASE) (EC 6.2.1.-) (Substrate--CoA ligase)
TCMDC-143669	Fatty acid synthase	Homo sapiens	200,171,937,792,763	349,168,425,608,292	O06586	Polyketide synthase (Probable polyketide synthase pks9)
TCMDC-143680	Fatty acid synthase	Homo sapiens	791,471,732,145,595	216,806,962,625,987	O06586	Polyketide synthase (Probable polyketide synthase pks9)
TCMDC-143682	Transketolase	Homo sapiens	887,616,660,042,559	250,278,250,821,521	O06811	Transketolase (TK) (EC 2.2.1.1)
TCMDC-143684	Luciferin 4-monooxygenase	Photuris pennsylvanica	159,655,040,138,576	237,473,084,117,749	O07169	PROBABLE FATTY-ACID-CoA LIGASE FADD7 (FATTY-ACID-CoA SYNTHETASE) (FATTY-ACID-CoA SYNTHASE) (EC 6.2.1.-)
TCMDC-143684	Luciferin 4-monooxygenase	Photuris pennsylvanica	159,655,040,138,576	237,473,084,117,749	O07411	PROBABLE FATTY-ACID-CoA LIGASE FADD5 (FATTY-ACID-CoA SYNTHETASE) (FATTY-ACID-CoA SYNTHASE) (EC 6.2.1.-)
TCMDC-143684	Monoglyceride lipase	Mus musculus	274,402,539,555,443	355,488,359,768,637	O07427	POSSIBLE LYSOPHOSPHOLIPASE (EC 3.1.-.-)
TCMDC-143684	Monoglyceride lipase	Mus musculus	21,932,059,933,903	304,935,916,597,201	O07427	POSSIBLE LYSOPHOSPHOLIPASE (EC 3.1.-.-)
TCMDC-143684	Monoglyceride lipase	Mus musculus	161,847,028,290,934	252,188,511,134,484	O07427	POSSIBLE LYSOPHOSPHOLIPASE (EC 3.1.-.-)
TCMDC-143684	Monoglyceride lipase	Mus musculus	143,867,304,402,923	235,687,295,554,283	O07427	POSSIBLE LYSOPHOSPHOLIPASE (EC 3.1.-.-)
TCMDC-143684	Monoglyceride lipase	Mus musculus	123,065,062,016,866	216,595,662,762,702	O07427	POSSIBLE LYSOPHOSPHOLIPASE (EC 3.1.-.-)

TCMDC-143698	D-amino-acid oxidase	Homo sapiens	117,220,410,666,063	256,401,281,192,378	O07727	Probable D-amino-acid oxidase (DAAO) (DAMOX) (DAO) (EC 1.4.3.3)
TCMDC-143698	SARS coronavirus 3C-like proteinase	SARS coronavirus	921,139,983,851,062	225,004,358,528,157	O07733	Uncharacterized protein Rv1899c/MT1950
TCMDC-143669	Fatty acid synthase	Homo sapiens	200,171,937,792,763	349,168,425,608,292	O07798	Phthioceranic/hydroxyphthioceranic acid synthase (EC 2.3.1.-) (Polyketide synthase pks2)
TCMDC-143680	Fatty acid synthase	Homo sapiens	791,471,732,145,595	216,806,962,625,987	O07798	Phthioceranic/hydroxyphthioceranic acid synthase (EC 2.3.1.-) (Polyketide synthase pks2)
TCMDC-143698	Glutathione reductase	Homo sapiens	163,628,870,495,036	309,028,608,024,495	O07927	Mycothione reductase (EC 1.8.1.15) (Mycothiol-disulfide reductase) (NADPH-dependent mycothione reductase)
TCMDC-143684	Glutathione reductase	Homo sapiens	11,226,472,108,928	244,213,697,039,155	O07927	Mycothione reductase (EC 1.8.1.15) (Mycothiol-disulfide reductase) (NADPH-dependent mycothione reductase)
TCMDC-143688	PROBABLE TRANSMEMBRANE CARBONIC ANHYDRASE (CARBONATE DEHYDRATASE) (CARBONIC DEHYDRATASE)	Mycobacterium tuberculosis	252,091,193,854,731	304,497,758,879,014	O33206	PROBABLE CONSERVED TRANSMEMBRANE PROTEIN (Sulfate transporter)
TCMDC-143698	PROBABLE TRANSMEMBRANE CARBONIC ANHYDRASE (CARBONATE DEHYDRATASE) (CARBONIC DEHYDRATASE)	Mycobacterium tuberculosis	15,872,594,107,475	227,855,770,813,936	O33206	PROBABLE CONSERVED TRANSMEMBRANE PROTEIN (Sulfate transporter)
TCMDC-143669	Fatty acid synthase	Homo sapiens	200,171,937,792,763	349,168,425,608,292	O50436	PROBABLE POLYKETIDE BETA-KETOACYL SYNTHASE PKS3 (EC 2.3.1.-)
TCMDC-143680	Fatty acid synthase	Homo sapiens	791,471,732,145,595	216,806,962,625,987	O50436	PROBABLE POLYKETIDE BETA-KETOACYL SYNTHASE PKS3 (EC 2.3.1.-)
TCMDC-143669	Fatty acid synthase	Homo sapiens	200,171,937,792,763	349,168,425,608,292	O50437	PROBABLE POLYKETIDE BETA-KETOACYL SYNTHASE PKS4 (EC 2.3.1.-)
TCMDC-143680	Fatty acid synthase	Homo sapiens	791,471,732,145,595	216,806,962,625,987	O50437	PROBABLE POLYKETIDE BETA-KETOACYL SYNTHASE PKS4 (EC 2.3.1.-)
TCMDC-143654	1-acylglycerol-3-phosphate O-acyltransferase beta	Homo sapiens	229,666,438,492,993	251,987,778,461,261	O53208	Acyltransferase family protein

TCMDC-143684	Luciferin 4-monooxygenase	Photuris pennsylvanica	159,655,040,138,576	237,473,084,117,749	O53306	PROBABLE CHAIN-FATTY-ACID-CoA LIGASE FADD13 (FATTY-ACYL-CoA SYNTHETASE) (EC 6.2.1.-) (Substrate--CoA ligase)
TCMDC-143684	Thymidine phosphorylase	Homo sapiens	108,180,587,005,845	228,564,208,820,063	O53366	Thymidine phosphorylase (EC 2.4.2.4) (TdRPase)
TCMDC-143669	Fatty acid synthase	Homo sapiens	200,171,937,792,763	349,168,425,608,292	O53490	Probable polyketide synthase pks12
TCMDC-143680	Fatty acid synthase	Homo sapiens	791,471,732,145,595	216,806,962,625,987	O53490	Probable polyketide synthase pks12
TCMDC-143698	Farnesyl diphosphate synthase	Homo sapiens	602,115,351,213,083	217,796,676,651,023	O53507	PROBABLE GERANYLGERANYL PYROPHOSPHATE SYNTHETASE IDSA2 (GGPPSASE) (GGPP SYNTHETASE) (GERANYLGERANYL DIPHOSPHATE SYNTHASE) (EC 2.5.1.-) (Polyprenyl synthetase)
TCMDC-143648	Farnesyl diphosphate synthase	Homo sapiens	458,205,372,980,296	205,730,986,723,994	O53507	PROBABLE GERANYLGERANYL PYROPHOSPHATE SYNTHETASE IDSA2 (GGPPSASE) (GGPP SYNTHETASE) (GERANYLGERANYL DIPHOSPHATE SYNTHASE) (EC 2.5.1.-) (Polyprenyl synthetase)
TCMDC-143662	Farnesyl pyrophosphate synthase	Leishmania donovani	328,807,127,310,695	203,727,871,258,663	O53507	PROBABLE GERANYLGERANYL PYROPHOSPHATE SYNTHETASE IDSA2 (GGPPSASE) (GGPP SYNTHETASE) (GERANYLGERANYL DIPHOSPHATE SYNTHASE) (EC 2.5.1.-) (Polyprenyl synthetase)
TCMDC-143654	1-acylglycerol-3-phosphate O-acyltransferase beta	Homo sapiens	229,666,438,492,993	251,987,778,461,261	O53516	1-acylglycerol-3-phosphate O-acyltransferase (EC 2.3.1.51) (Acyltransferase family protein)
TCMDC-143688	Carbonic anhydrase	Saccharomyces cerevisiae S288c	275,376,529,005,328	507,317,213,149,951	O53573	Carbonic anhydrase (EC 4.2.1.1) (Carbonate dehydratase)
TCMDC-143698	Carbonic anhydrase	Saccharomyces cerevisiae S288c	271,883,148,008,429	502,155,501,671,741	O53573	Carbonic anhydrase (EC 4.2.1.1) (Carbonate dehydratase)
TCMDC-143698	Carbonic anhydrase 1	Helicobacter pylori (strain ATCC 700392 / 26695) (Campylobacterpylori)	300,843,288,048,303	430,428,994,460,207	O53573	Carbonic anhydrase (EC 4.2.1.1) (Carbonate dehydratase)

TCMDC-143688	Carbonic anhydrase 1	Helicobacter pylori (strain ATCC 700392 / 26695) (Campylobacterpylori)	269,638,914,751,048	396,474,730,769,485	O53573	Carbonic anhydrase (EC 4.2.1.1) (Carbonate dehydratase)
TCMDC-143648	Carbonic anhydrase 1	Helicobacter pylori (strain ATCC 700392 / 26695) (Campylobacterpylori)	220,877,940,162,516	343,416,687,487,415	O53573	Carbonic anhydrase (EC 4.2.1.1) (Carbonate dehydratase)
TCMDC-143648	Carbonic anhydrase	Saccharomyces cerevisiae S288c	149,633,877,118,092	321,523,768,311,528	O53573	Carbonic anhydrase (EC 4.2.1.1) (Carbonate dehydratase)
TCMDC-143698	Carbonic anhydrase	Candida albicans (strain SC5314 / ATCC MYA-2876) (Yeast)	208,747,149,467,744	285,487,222,215,394	O53573	Carbonic anhydrase (EC 4.2.1.1) (Carbonate dehydratase)
TCMDC-143688	Carbonic anhydrase	Candida albicans (strain SC5314 / ATCC MYA-2876) (Yeast)	182,617,277,115,469	263,228,891,063,653	O53573	Carbonic anhydrase (EC 4.2.1.1) (Carbonate dehydratase)
TCMDC-143684	Carbonic anhydrase	Saccharomyces cerevisiae S288c	944,658,531,286,181	240,009,206,083,868	O53573	Carbonic anhydrase (EC 4.2.1.1) (Carbonate dehydratase)
TCMDC-143698	Carbonic anhydrase	Saccharomyces cerevisiae S288c	91,258,274,352,607	235,269,787,108,355	O53573	Carbonic anhydrase (EC 4.2.1.1) (Carbonate dehydratase)
TCMDC-143698	Carbonic anhydrase 1	Helicobacter pylori (strain ATCC 700392 / 26695) (Campylobacterpylori)	110,983,246,117,346	223,837,505,932,464	O53573	Carbonic anhydrase (EC 4.2.1.1) (Carbonate dehydratase)
TCMDC-143654	Carbonic anhydrase 1	Helicobacter pylori (strain ATCC 700392 / 26695) (Campylobacterpylori)	921,765,050,520,944	20,337,341,779,938	O53573	Carbonic anhydrase (EC 4.2.1.1) (Carbonate dehydratase)
TCMDC-143669	Fatty acid synthase	Homo sapiens	200,171,937,792,763	349,168,425,608,292	O53579	POLYKETIDE SYNTHASE PKS13 (Polyketide synthase)
TCMDC-143680	Fatty acid synthase	Homo sapiens	791,471,732,145,595	216,806,962,625,987	O53579	POLYKETIDE SYNTHASE PKS13 (Polyketide synthase)

TCMDC-143648	P-glycoprotein 1	Mus musculus	135,682,881,209,571	219,521,823,829,612	O53645	PROBABLE DRUGS-TRANSPORT TRANSMEMBRANE ATP-BINDING PROTEIN ABC TRANSPORTER
TCMDC-143698	GAR transformylase	Homo sapiens	7,426,129,699,716	238,171,840,641,907	O53823	Phosphoribosylformylglycinamidine cyclo-ligase (EC 6.3.3.1)
TCMDC-143669	Fatty acid synthase	Homo sapiens	200,171,937,792,763	349,168,425,608,292	O53901	Probable polyketide synthase pks5
TCMDC-143680	Fatty acid synthase	Homo sapiens	791,471,732,145,595	216,806,962,625,987	O53901	Probable polyketide synthase pks5
TCMDC-143669	Fatty acid synthase	Homo sapiens	200,171,937,792,763	349,168,425,608,292	O65933	Probable polyketide synthase pks8
TCMDC-143680	Fatty acid synthase	Homo sapiens	791,471,732,145,595	216,806,962,625,987	O65933	Probable polyketide synthase pks8
TCMDC-143669	Fatty acid synthase	Homo sapiens	200,171,937,792,763	349,168,425,608,292	O86335	PROBABLE MEMBRANE BOUND POLYKETIDE SYNTHASE PKS6
TCMDC-143680	Fatty acid synthase	Homo sapiens	791,471,732,145,595	216,806,962,625,987	O86335	PROBABLE MEMBRANE BOUND POLYKETIDE SYNTHASE PKS6
TCMDC-143682	Methionine aminopeptidase 1	Saccharomyces cerevisiae S288c	415,538,225,528,142	463,867,870,982,404	P0A5J2	Methionine aminopeptidase 2 (MAP) (EC 3.4.11.18) (Peptidase M)
TCMDC-143682	Methionine aminopeptidase	Escherichia coli K-12	470,090,032,443,634	405,963,752,649,259	P0A5J2	Methionine aminopeptidase 2 (MAP) (EC 3.4.11.18) (Peptidase M)
TCMDC-143669	Methionine aminopeptidase	Escherichia coli K-12	303,707,733,899,713	308,537,330,026,973	P0A5J2	Methionine aminopeptidase 2 (MAP) (EC 3.4.11.18) (Peptidase M)
TCMDC-143648	Methionine aminopeptidase 1	Saccharomyces cerevisiae S288c	123,684,806,771,458	249,827,980,008,733	P0A5J2	Methionine aminopeptidase 2 (MAP) (EC 3.4.11.18) (Peptidase M)
TCMDC-143648	Methionine aminopeptidase 1	Saccharomyces cerevisiae S288c	861,228,035,422,242	222,280,702,645,364	P0A5J2	Methionine aminopeptidase 2 (MAP) (EC 3.4.11.18) (Peptidase M)
TCMDC-143648	Methionine aminopeptidase	Escherichia coli K-12	123,942,410,699,144	203,274,375,348,252	P0A5J2	Methionine aminopeptidase 2 (MAP) (EC 3.4.11.18) (Peptidase M)
TCMDC-143698	Methionine aminopeptidase 1	Homo sapiens	780,610,066,544,102	201,480,715,915,867	P0A5J2	Methionine aminopeptidase 2 (MAP) (EC 3.4.11.18) (Peptidase M)
TCMDC-143654	Dihydrofolate reductase	Saccharomyces cerevisiae S288c	588,819,257,641,712	207,097,020,319,096	P0A546	Dihydrofolate reductase (EC 1.5.1.3)
TCMDC-143698	Taq polymerase 1	Thermus aquaticus	724,590,745,807,126	209,600,451,559,606	P0A550	DNA polymerase I (POL I) (EC 2.7.7.7)
TCMDC-143684	Monoamine oxidase B	Bos taurus	234,394,426,729,223	277,741,403,723,271	P63533	Putative flavin-containing monoamine oxidase AofH (EC 1.4.3.-)
TCMDC-	Monoamine oxidase A	Bos taurus	143,005,003,950,058	224,927,298,249,012	P63533	Putative flavin-containing monoamine

143684						oxidase AofH (EC 1.4.3.-)
TCMDC-143688	Uncharacterized protein Rv1284/MT1322	Mycobacterium tuberculosis	275,710,879,362,787	327,392,328,315,567	P64797	Putative carbonate dehydratase-like protein Rv1284 (EC 4.2.1.-)
TCMDC-143698	Uncharacterized protein Rv1284/MT1322	Mycobacterium tuberculosis	207,218,552,602,899	270,218,238,746,845	P64797	Putative carbonate dehydratase-like protein Rv1284 (EC 4.2.1.-)
TCMDC-143648	Uncharacterized protein Rv1284/MT1322	Mycobacterium tuberculosis	131,510,222,213,344	207,020,577,526,118	P64797	Putative carbonate dehydratase-like protein Rv1284 (EC 4.2.1.-)
TCMDC-143698	GAR transformylase	Homo sapiens	7,426,129,699,716	238,171,840,641,907	P65893	Phosphoribosylamine--glycine ligase (EC 6.3.4.13) (GARS) (Glycinamide ribonucleotide synthetase) (Phosphoribosylglycinamide synthetase)
TCMDC-143684	Acetyl-CoA acetyltransferase, mitochondrial	Homo sapiens	904,915,537,137,182	226,198,959,812,069	P66926	Probable acetyl-CoA acetyltransferase (EC 2.3.1.9) (Acetoacetyl-CoA thiolase)
TCMDC-143678	Lysyl-tRNA synthetase	Homo sapiens	216,194,196,301,634	267,798,020,940,938	P67607	Lysine--tRNA ligase 1 (EC 6.1.1.6) (Lysyl-tRNA synthetase 1) (LysRS 1)
TCMDC-143688	S-adenosylhomocysteine nucleosidase	Escherichia coli K-12	126,449,351,515,014	276,773,384,605,152	P67656	MTA/SAH nucleosidase (EC 3.2.2.9) (5'-methylthioadenosine nucleosidase) (S-adenosylhomocysteine nucleosidase)
TCMDC-143698	S-adenosylhomocysteine nucleosidase	Escherichia coli K-12	99,898,427,747,966	254,697,450,092,595	P67656	MTA/SAH nucleosidase (EC 3.2.2.9) (5'-methylthioadenosine nucleosidase) (S-adenosylhomocysteine nucleosidase)
TCMDC-143654	S-adenosylhomocysteine nucleosidase	Escherichia coli K-12	704,106,723,948,018	23,017,966,827,038	P67656	MTA/SAH nucleosidase (EC 3.2.2.9) (5'-methylthioadenosine nucleosidase) (S-adenosylhomocysteine nucleosidase)
TCMDC-143698	S-adenosylhomocysteine nucleosidase	Escherichia coli K-12	374,347,426,232,409	202,761,621,632,353	P67656	MTA/SAH nucleosidase (EC 3.2.2.9) (5'-methylthioadenosine nucleosidase) (S-adenosylhomocysteine nucleosidase)
TCMDC-143698	Beta-lactamase	Pseudomonas aeruginosa (strain ATCC 15692 / PAO1 / 1C / PRS 101 / LMG12228)	602,811,186,137,248	214,122,767,835,343	P71988	POSSIBLE PENICILLIN-BINDING PROTEIN
TCMDC-143678	Lysyl-tRNA synthetase	Homo sapiens	216,194,196,301,634	267,798,020,940,938	P94974	Lysylphosphatidylglycerol biosynthesis bifunctional protein LysX [Includes: Lysine--tRNA ligase (EC 6.1.1.6) (Lysyl-tRNA synthetase) (LysRS); Phosphatidylglycerol lysyltransferase (EC 2.3.2.3) (Lysylphosphatidylglycerol

						synthetase) (LPG synthetase)]
TCMDC-143669	Fatty acid synthase	Homo sapiens	200,171,937,792,763	349,168,425,608,292	P94996	Polyketide synthase (Probable polyketide synthase pks7)
TCMDC-143680	Fatty acid synthase	Homo sapiens	791,471,732,145,595	216,806,962,625,987	P94996	Polyketide synthase (Probable polyketide synthase pks7)
TCMDC-143684	Aldose reductase	Sus scrofa	782,248,513,399,084	2,036,739,455,885	P95124	Uncharacterized oxidoreductase Rv2971/MT3049 (EC 1.-.-.-)
TCMDC-143684	Luciferin 4-monooxygenase	Photuris pennsylvanica	159,655,040,138,576	237,473,084,117,749	P95213	Long-chain-fatty-acid--AMP ligase FadD30 (FAAL) (EC 6.2.1.-) (Acyl-AMP synthetase)
TCMDC-143684	Luciferin 4-monooxygenase	Photuris pennsylvanica	159,655,040,138,576	237,473,084,117,749	P95227	PROBABLE FATTY-ACID-CoA LIGASE FADD2 (FATTY-ACID-CoA SYNTHETASE) (FATTY-ACID-CoA SYNTHASE) (EC 6.2.1.-) (Substrate--CoA ligase)
TCMDC-143669	Fatty acid synthase	Homo sapiens	200,171,937,792,763	349,168,425,608,292	P96202	Phthiocerol synthesis polyketide synthase type I PpsC (Beta-ketoacyl-acyl-carrier-protein synthase I) (EC 2.3.1.41)
TCMDC-143680	Fatty acid synthase	Homo sapiens	791,471,732,145,595	216,806,962,625,987	P96202	Phthiocerol synthesis polyketide synthase type I PpsC (Beta-ketoacyl-acyl-carrier-protein synthase I) (EC 2.3.1.41)
TCMDC-143669	Fatty acid synthase	Homo sapiens	200,171,937,792,763	349,168,425,608,292	P96203	Phthiocerol synthesis polyketide synthase type I PpsD (Beta-ketoacyl-acyl-carrier-protein synthase I) (EC 2.3.1.41)
TCMDC-143680	Fatty acid synthase	Homo sapiens	791,471,732,145,595	216,806,962,625,987	P96203	Phthiocerol synthesis polyketide synthase type I PpsD (Beta-ketoacyl-acyl-carrier-protein synthase I) (EC 2.3.1.41)
TCMDC-143669	Fatty acid synthase	Homo sapiens	200,171,937,792,763	349,168,425,608,292	P96204	Phthiocerol synthesis polyketide synthase type I PpsE (Beta-ketoacyl-acyl-carrier-protein synthase I) (EC 2.3.1.41)
TCMDC-143680	Fatty acid synthase	Homo sapiens	791,471,732,145,595	216,806,962,625,987	P96204	Phthiocerol synthesis polyketide synthase type I PpsE (Beta-ketoacyl-acyl-carrier-protein synthase I) (EC 2.3.1.41)
TCMDC-143684	HTH-type transcriptional regulator EthR	Mycobacterium tuberculosis	113,100,255,413,274	237,297,914,700,054	P96222	HTH-type transcriptional regulator EthR
TCMDC-143684	HTH-type transcriptional regulator EthR	Mycobacterium tuberculosis	102,284,922,859,784	22,694,469,322,272	P96222	HTH-type transcriptional regulator EthR
TCMDC-143648	HTH-type transcriptional regulator EthR	Mycobacterium tuberculosis	929,831,982,613,761	21,804,040,716,289	P96222	HTH-type transcriptional regulator EthR
TCMDC-	HTH-type transcriptional	Mycobacterium	882,553,723,856,707	213,514,589,010,247	P96222	HTH-type transcriptional regulator EthR

143680	regulator EthR	tuberculosis				
TCMDC-143682	HTH-type transcriptional regulator EthR	Mycobacterium tuberculosis	782,314,948,236,383	203,919,005,707,734	P96222	HTH-type transcriptional regulator EthR
TCMDC-143698	DNA-(apurinic or apyrimidinic site) lyase	Homo sapiens	129,308,693,493,425	236,946,658,451,883	P96273	Exodeoxyribonuclease III (EC 3.1.11.2) (PROBABLE EXODEOXYRIBONUCLEASE III PROTEIN XTHA (EXONUCLEASE III) (EXO III) (AP ENDONUCLEASE VI)) (EC 3.1.11.2)
TCMDC-143698	Peptide deformylase	Pseudomonas aeruginosa (strain ATCC 15692 / PAO1 / 1C / PRS 101 / LMG12228)	105,187,569,264,415	24,440,187,923,122	P96275	Peptide deformylase (PDF) (EC 3.5.1.88) (Polypeptide deformylase)
TCMDC-143684	Peptide deformylase	Escherichia coli K-12	879,090,670,534,146	232,767,021,850,308	P96275	Peptide deformylase (PDF) (EC 3.5.1.88) (Polypeptide deformylase)
TCMDC-143654	Transitional endoplasmic reticulum ATPase	Homo sapiens	121,940,668,229,232	233,862,058,406,166	P96281	Cell division control protein, putative (PUTATIVE CONSERVED ATPASE) (EC 3.6.1.-)
TCMDC-143698	Transitional endoplasmic reticulum ATPase	Homo sapiens	119,660,833,874,604	231,699,288,484,281	P96281	Cell division control protein, putative (PUTATIVE CONSERVED ATPASE) (EC 3.6.1.-)
TCMDC-143678	Transitional endoplasmic reticulum ATPase	Homo sapiens	92,376,186,429,171	205,815,647,111,632	P96281	Cell division control protein, putative (PUTATIVE CONSERVED ATPASE) (EC 3.6.1.-)
TCMDC-143669	Fatty acid synthase	Homo sapiens	200,171,937,792,763	349,168,425,608,292	P96284	Putative inactive phenolphthiocerol synthesis polyketide synthase type I Pks15
TCMDC-143680	Fatty acid synthase	Homo sapiens	791,471,732,145,595	216,806,962,625,987	P96284	Putative inactive phenolphthiocerol synthesis polyketide synthase type I Pks15
TCMDC-143669	Fatty acid synthase	Homo sapiens	200,171,937,792,763	349,168,425,608,292	P96285	Putative inactive phenolphthiocerol synthesis polyketide synthase type I Pks1
TCMDC-143680	Fatty acid synthase	Homo sapiens	791,471,732,145,595	216,806,962,625,987	P96285	Putative inactive phenolphthiocerol synthesis polyketide synthase type I Pks1
TCMDC-143669	Fatty acid synthase	Homo sapiens	200,171,937,792,763	349,168,425,608,292	P96291	Mycocerosic acid synthase (PROBABLE MULTIFUNCTIONAL MYCOCEROSIC ACID SYNTHASE MEMBRANE-ASSOCIATED MAS)
TCMDC-143680	Fatty acid synthase	Homo sapiens	791,471,732,145,595	216,806,962,625,987	P96291	Mycocerosic acid synthase (PROBABLE MULTIFUNCTIONAL MYCOCEROSIC ACID SYNTHASE MEMBRANE-ASSOCIATED MAS)
TCMDC-143684	Luciferin 4-monooxygenase	Photuris pennsylvanica	159,655,040,138,576	237,473,084,117,749	P96843	PROBABLE FATTY-ACID-CoA LIGASE FADD3 (FATTY-ACID-CoA SYNTHETASE) (FATTY-ACID-CoA SYNTHASE) (EC 6.2.1.-)

TCMDC-143684	Monoacylglycerol lipase ABHD6	Mus musculus	296,002,005,186,462	386,738,136,554,808	P96851	4,5-9,10-diseco-3-hydroxy-5,9,17-trioxoandrosta-1(10),2-diene-4-oate hydrolase (2-hydroxy-6-oxo-6-phenylhexa-2,4-dienoate hydrolase) (HOPDA hydrolase) (EC 3.7.1.8) (Meta-cleavage product hydrolase) (MCP hydrolase)
TCMDC-143684	Monoacylglycerol lipase ABHD6	Mus musculus	250,664,348,257,219	343,835,435,380,042	P96851	4,5-9,10-diseco-3-hydroxy-5,9,17-trioxoandrosta-1(10),2-diene-4-oate hydrolase (2-hydroxy-6-oxo-6-phenylhexa-2,4-dienoate hydrolase) (HOPDA hydrolase) (EC 3.7.1.8) (Meta-cleavage product hydrolase) (MCP hydrolase)
TCMDC-143684	Monoacylglycerol lipase ABHD6	Mus musculus	189,010,681,795,267	285,493,010,832,741	P96851	4,5-9,10-diseco-3-hydroxy-5,9,17-trioxoandrosta-1(10),2-diene-4-oate hydrolase (2-hydroxy-6-oxo-6-phenylhexa-2,4-dienoate hydrolase) (HOPDA hydrolase) (EC 3.7.1.8) (Meta-cleavage product hydrolase) (MCP hydrolase)
TCMDC-143684	Monoacylglycerol lipase ABHD6	Mus musculus	16,107,809,476,367	259,060,603,081,167	P96851	4,5-9,10-diseco-3-hydroxy-5,9,17-trioxoandrosta-1(10),2-diene-4-oate hydrolase (2-hydroxy-6-oxo-6-phenylhexa-2,4-dienoate hydrolase) (HOPDA hydrolase) (EC 3.7.1.8) (Meta-cleavage product hydrolase) (MCP hydrolase)
TCMDC-143684	Monoacylglycerol lipase ABHD6	Mus musculus	152,634,204,156,067	251,070,209,663,709	P96851	4,5-9,10-diseco-3-hydroxy-5,9,17-trioxoandrosta-1(10),2-diene-4-oate hydrolase (2-hydroxy-6-oxo-6-phenylhexa-2,4-dienoate hydrolase) (HOPDA hydrolase) (EC 3.7.1.8) (Meta-cleavage product hydrolase) (MCP hydrolase)
TCMDC-143688	PROBABLE TRANSMEMBRANE CARBONIC ANHYDRASE	Mycobacterium tuberculosis	252,091,193,854,731	304,497,758,879,014	P96878	PROBABLE TRANSMEMBRANE CARBONIC ANHYDRASE (CARBONATE DEHYDRATASE) (CARBONIC DEHYDRATASE)

	(CARBONATE DEHYDRATASE) (CARBONIC DEHYDRATASE)					(EC 4.2.1.1) (Sulfate transporter/carbonic anhydrase, putative)
TCMDC-143698	PROBABLE TRANSMEMBRANE CARBONIC ANHYDRASE (CARBONATE DEHYDRATASE) (CARBONIC DEHYDRATASE)	Mycobacterium tuberculosis	15,872,594,107,475	227,855,770,813,936	P96878	PROBABLE TRANSMEMBRANE CARBONIC ANHYDRASE (CARBONATE DEHYDRATASE) (CARBONIC DEHYDRATASE) (EC 4.2.1.1) (Sulfate transporter/carbonic anhydrase, putative)
TCMDC-143682	Methionine aminopeptidase 1	Saccharomyces cerevisiae S288c	415,538,225,528,142	463,867,870,982,404	Q7D9D5	Methionine aminopeptidase 1 (MAP) (EC 3.4.11.18) (Peptidase M)
TCMDC-143682	Methionine aminopeptidase	Escherichia coli K-12	470,090,032,443,634	405,963,752,649,259	Q7D9D5	Methionine aminopeptidase 1 (MAP) (EC 3.4.11.18) (Peptidase M)
TCMDC-143669	Methionine aminopeptidase	Escherichia coli K-12	303,707,733,899,713	308,537,330,026,973	Q7D9D5	Methionine aminopeptidase 1 (MAP) (EC 3.4.11.18) (Peptidase M)
TCMDC-143648	Methionine aminopeptidase 1	Saccharomyces cerevisiae S288c	123,684,806,771,458	249,827,980,008,733	Q7D9D5	Methionine aminopeptidase 1 (MAP) (EC 3.4.11.18) (Peptidase M)
TCMDC-143648	Methionine aminopeptidase 1	Saccharomyces cerevisiae S288c	861,228,035,422,242	222,280,702,645,364	Q7D9D5	Methionine aminopeptidase 1 (MAP) (EC 3.4.11.18) (Peptidase M)
TCMDC-143648	Methionine aminopeptidase	Escherichia coli K-12	123,942,410,699,144	203,274,375,348,252	Q7D9D5	Methionine aminopeptidase 1 (MAP) (EC 3.4.11.18) (Peptidase M)
TCMDC-143698	Methionine aminopeptidase 1	Homo sapiens	780,610,066,544,102	201,480,715,915,867	Q7D9D5	Methionine aminopeptidase 1 (MAP) (EC 3.4.11.18) (Peptidase M)
TCMDC-143682	M17 leucyl aminopeptidase	Plasmodium falciparum 3D7	275,320,435,854,952	212,171,170,238,322	Q10401	Probable cytosol aminopeptidase (EC 3.4.11.1) (Leucine aminopeptidase) (LAP) (EC 3.4.11.10) (Leucyl aminopeptidase)
TCMDC-143669	Fatty acid synthase	Homo sapiens	200,171,937,792,763	349,168,425,608,292	Q10977	Phthiocerol synthesis polyketide synthase type I PpsA (Beta-ketoacyl-acyl-carrier-protein synthase I) (EC 2.3.1.41)
TCMDC-143680	Fatty acid synthase	Homo sapiens	791,471,732,145,595	216,806,962,625,987	Q10977	Phthiocerol synthesis polyketide synthase type I PpsA (Beta-ketoacyl-acyl-carrier-protein synthase I) (EC 2.3.1.41)
TCMDC-143669	Fatty acid synthase	Homo sapiens	200,171,937,792,763	349,168,425,608,292	Q10978	Phthiocerol synthesis polyketide synthase type I PpsB (Beta-ketoacyl-acyl-carrier-protein synthase I) (EC 2.3.1.41)
TCMDC-143680	Fatty acid synthase	Homo sapiens	791,471,732,145,595	216,806,962,625,987	Q10978	Phthiocerol synthesis polyketide synthase type I PpsB (Beta-ketoacyl-acyl-carrier-protein synthase I) (EC 2.3.1.41)

Supplemental References

1. Fernandez P, Saint-Joanis B, Barilone N, Jackson M, Gicquel B, Cole ST, et al. The Ser/Thr protein kinase PknB is essential for sustaining mycobacterial growth. Journal of bacteriology. 2006;188(22):7778-84.
2. Murphy DJ, Brown JR. Identification of gene targets against dormant phase Mycobacterium tuberculosis infections. BMC infectious diseases. 2007;7:84.
3. Sassetti CM, Boyd DH, Rubin EJ. Genes required for mycobacterial growth defined by high density mutagenesis. Molecular microbiology. 2003;48(1):77-84.
4. Wehenkel A, Bellinzoni M, Grana M, Duran R, Villarino A, Fernandez P, et al. Mycobacterial Ser/Thr protein kinases and phosphatases: physiological roles and therapeutic potential. Biochimica et biophysica acta. 2008;1784(1):193-202.
5. Venos ES, Knodel MH, Radford CL, Berger BJ. Branched-chain amino acid aminotransferase and methionine formation in Mycobacterium tuberculosis. BMC microbiology. 2004;4:39.
6. Noy T, Xu H, Blanchard JS. Acetylation of acetyl-CoA synthetase from Mycobacterium tuberculosis leads to specific inactivation of the adenylation reaction. Archives of biochemistry and biophysics. 2014;550-551:42-9.
7. Xu H, Hegde SS, Blanchard JS. Reversible acetylation and inactivation of Mycobacterium tuberculosis acetyl-CoA synthetase is dependent on cAMP. Biochemistry. 2011;50(26):5883-92.
8. Gupta AK, Katoch VM, Chauhan DS, Sharma R, Singh M, Venkatesan K, et al. Microarray analysis of efflux pump genes in multidrug-resistant Mycobacterium tuberculosis during stress induced by common anti-tuberculous drugs. Microbial drug resistance. 2010;16(1):21-8.
9. Martinez-Jimenez F, Papadatos G, Yang L, Wallace IM, Kumar V, Pieper U, et al. Target prediction for an open access set of compounds active against Mycobacterium tuberculosis. PLoS computational biology. 2013;9(10):e1003253.
10. Ballell L, Bates RH, Young RJ, Alvarez-Gomez D, Alvarez-Ruiz E, Barroso V, et al. Fueling open-source drug discovery: 177 small-molecule leads against tuberculosis. ChemMedChem. 2013;8(2):313-21.
11. Altschul SF, Madden TL, Schaffer AA, Zhang J, Zhang Z, Miller W, et al. Gapped BLAST and PSI-BLAST: a new generation of protein database search programs. Nucleic acids research. 1997;25(17):3389-402.
